# Activation of SLIT2/ROBO1/LRP6 axis aggravates cartilage degradation via **β**-catenin signaling in TMJOA

**DOI:** 10.1172/jci.insight.193632

**Published:** 2026-04-08

**Authors:** Guan Luo, Baoyi Chen, Wenjun Chen, Huiyi Lin, Weiqi Guo, Qingbin Zhang, Jiang Li, Lijing Wang, Janak Lal Pathak, Yuhui Yang, Weijun Zhang, Xiaoyu Zhang, Beining Zheng, Ziyi Wang, Shiting Wei, Jiaxin He, Wei-Jie Zhou, Chang Liu

**Affiliations:** 1 Department of Orthodontics, School and Hospital of Stomatology, Guangdong Engineering Research Center of Oral Restoration and Reconstruction, Guangzhou Key Laboratory of Basic and Applied Research of Oral Regenerative Medicine, Guangzhou Medical University, Guangzhou, Guangdong, China.; 2 Department of Orthodontics, Jiangmen Municipal Stomatology Hospital, Jiangmen, Guangdong, China.; 3 Department of Stomatology, The First Affiliated Hospital of Guangdong Pharmaceutical University, Guangzhou, Guangdong, China.; 4 Vascular Biology Research Institute, School of Life Sciences and Biopharmaceutics, Guangdong Pharmaceutical University, Guangzhou, Guangdong, China.; 5 Department of Orthopedics, Guangdong Provincial People’s Hospital (Guangdong Academy of Medical Sciences), Southern Medical University, Guangzhou, Guangdong, China.; 6 Department of General Surgery, Guangdong Provincial Key Laboratory of Precision Medicine for Gastrointestinal Tumor, State Key Laboratory of Multi-organ Injury Prevention and Treatment, Cancer Research Institute, School of Basic Medical Sciences, Nanfang Hospital, Southern Medical University, Guangzhou, Guangdong, China.

**Keywords:** Bone biology, Inflammation, Arthritis, Cartilage, Signal transduction

## Abstract

Temporomandibular joint osteoarthritis (TMJOA), a prevalent subtype of temporomandibular disorders, is characterized by progressive cartilage degradation and subchondral bone destruction. Despite advancements in understanding TMJOA pathogenesis, the molecular mechanisms underlying its progression remain unclear. In this study, elevated Slit guidance ligand 2 (SLIT2) expression was observed in TMJ tissues of unilateral anterior crossbite–induced TMJOA mice and synovial fluid from patients with TMJOA, correlating with disease severity. Furthermore, SLIT2 overexpression in transgenic mice intensified TMJOA progression, whereas heterozygous deletion of roundabout guidance receptor 1/2 (ROBO1/2) alleviated cartilage and bone damage. Mechanistically, SLIT2 promoted ROBO1-LRP6 complex formation, facilitating LRP6 phosphorylation and β-catenin nuclear translocation. This cascade upregulated matrix-degrading enzymes while downregulating cartilage structural proteins, exacerbating cartilage destruction and subchondral bone loss. These findings suggest that the SLIT2/ROBO1/LRP6 axis may represent a potential therapeutic target for TMJOA and provide mechanistic insights into disease progression.

## Abstract 2

This study reveals how the SLIT2/ROBO1/LRP6 axis exacerbates cartilage degradation in TMJ osteoarthritis through β-catenin signaling.

## Graphical abstract

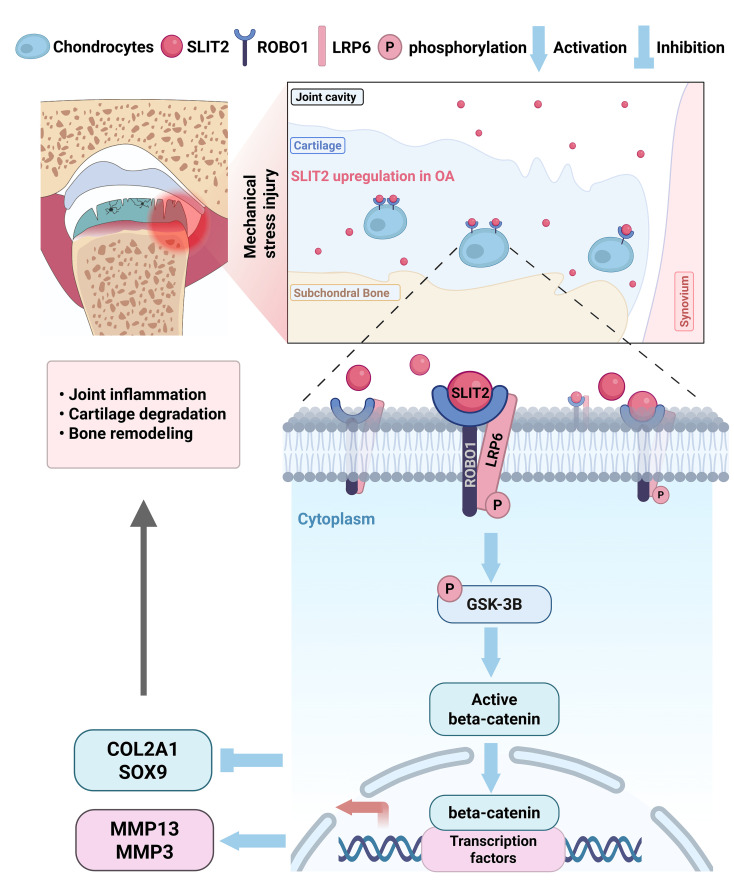

## Introduction

Temporomandibular joint osteoarthritis (TMJOA), a common variant of temporomandibular disorders (TMDs), often results from trauma, condylar overload, or developmental anomalies (1, 2). Proinflammatory cytokines like IL-1β and TNF-α accelerate matrix degradation and hinder tissue repair (3). However, the underlying inflammatory mechanisms of TMJOA remain unclear. Current osteoarthritis (OA) therapies are limited and inadequate for preventing the initiation and progression of the disease (4, 5).

Slit guidance ligand 2 (SLIT2) is a secreted protein that interacts with roundabout guidance receptors 1–4 (ROBO1–4) (6, 7). The second leucine-rich-repeat (LRR) domain of SLIT2 interacts with the first immunoglobulin-like domain of ROBOs, enabling ROBO to interact with specific adaptor proteins to activate downstream signaling (8, 9). SLIT/ROBO signaling was initially identified in the neurological system, where it functions as an axon guidance molecule (10, 11). Subsequently, the expression of SLITs and ROBOs was observed in multiple regions of the developing vertebrate embryo, including the heart, kidney, branchial arches (I and II), and limb bud, especially in and around the developing joints (12, 13). Recently, dysregulation of the SLIT2/ROBO pathway has been associated with the progression of various diseases. SLIT2 expression was aberrantly elevated in neurons and astrocytes in neuropsychiatric disorders, such as Alzheimer disease and temporal lobe epilepsy (14, 15). Patients with lupus nephritis exhibited elevated blood SLIT2 levels and increased expression of ROBO1 and SLIT2 in renal tubular epithelial cells (16). Additionally, alveolar bone loss and inflammatory reactions were made worse by the overexpression of SLIT2 in periodontitis (17, 18). It has been suggested that the function of SLIT2/ROBO signaling is highly context and cell specific; however, the involvement of SLIT2/ROBO signaling in TMJOA remains poorly understood. Recent studies have shown that Netrin-1, an axon guidance molecule functionally similar to SLIT2, is elevated in the synovial fluid of patients and correlates with pain severity (19). In addition, inhibition of SLIT3/RORO1 signaling has been reported to alleviate subchondral bone destruction in OA, further underscoring the pathological relevance of SLIT/ROBO pathway dysregulation (20). The involvement of the SLIT2/ROBO1 pathway in the pathogenesis and progression of TMJOA requires further investigation.

The β-catenin protein is crucial for the formation of adherens junction complexes mediated by E-cadherin and for canonical Wnt signaling (21, 22). Our prior research indicated that the SLIT2/ROBO1 signaling pathway diminishes β-catenin–E-cadherin complex formation, releasing β-catenin from the complex (23, 24). Nonetheless, the mechanistic connection between SLIT2/ROBO1 signaling and the canonical Wnt/β-catenin pathway is inadequately defined. The canonical Wnt/β-catenin signaling pathway handles multiple cellular processes, such as cell proliferation, differentiation, and inflammation (25). Wnt ligand binding to Frizzled receptors and coreceptor low-density lipoprotein receptor–related protein 6 (LRP6) activates the pathway, causing LRP6 phosphorylation, inhibiting glycogen synthase kinase-3β (GSK-3β) activity, and stabilizing β-catenin. Following β-catenin accumulation in the cytoplasm, it translocates to the nucleus to activate target gene transcription (26, 27). A mouse model expressing constitutively active β-catenin showed OA-like symptoms, including cartilage loss and osteophyte production, due to elevated β-catenin levels in articular chondrocyte nuclei (28). Our earlier findings revealed that SLIT2 synergistically amplifies β-catenin activation mediated by the Wnt agonist R-spondin-1 in a ROBO1-dependent manner (29), highlighting a potential interaction between SLIT2/ROBO1 signaling and the canonical Wnt/β-catenin pathway. Binnerts et al. further demonstrated that R-spondin-1 potentiates β-catenin signaling through enhanced LRP6 membrane localization rather than direct receptor activation (30). Taken together, these data support the hypothesis that SLIT2/ROBO1 signaling amplifies β-catenin activation via an LRP6-mediated mechanism. Specifically, we hypothesized that SLIT2/ROBO1 facilitates LRP6 phosphorylation, thereby enhancing downstream β-catenin nuclear translocation and transcriptional activity. Clarifying the upstream regulatory mechanisms of β-catenin would help to identify potential therapeutic targets and inform the development of treatment strategies for TMJOA.

In this study, we observed increased SLIT2 levels in TMJ lavage fluid of patients with TMJOA and in the cartilage of mice with unilateral anterior crossbite–induced (UAC-induced) TMJOA. Additionally, we showed that SLIT2 augmented the interaction between ROBO1 and LRP6, resulting in increased phosphorylation of LRP6, and that this increases the nuclear translocation of β-catenin and worsens cartilage destruction in TMJOA. Our findings implicate the SLIT2/ROBO1/LRP6 complex in TMJOA pathogenesis and emphasize its role in the β-catenin signaling, potentially contributing to developing targeted therapeutic strategies.

## Results

### Upregulation of SLIT2 expression in joint tissues in OA.

**Figure 1:**
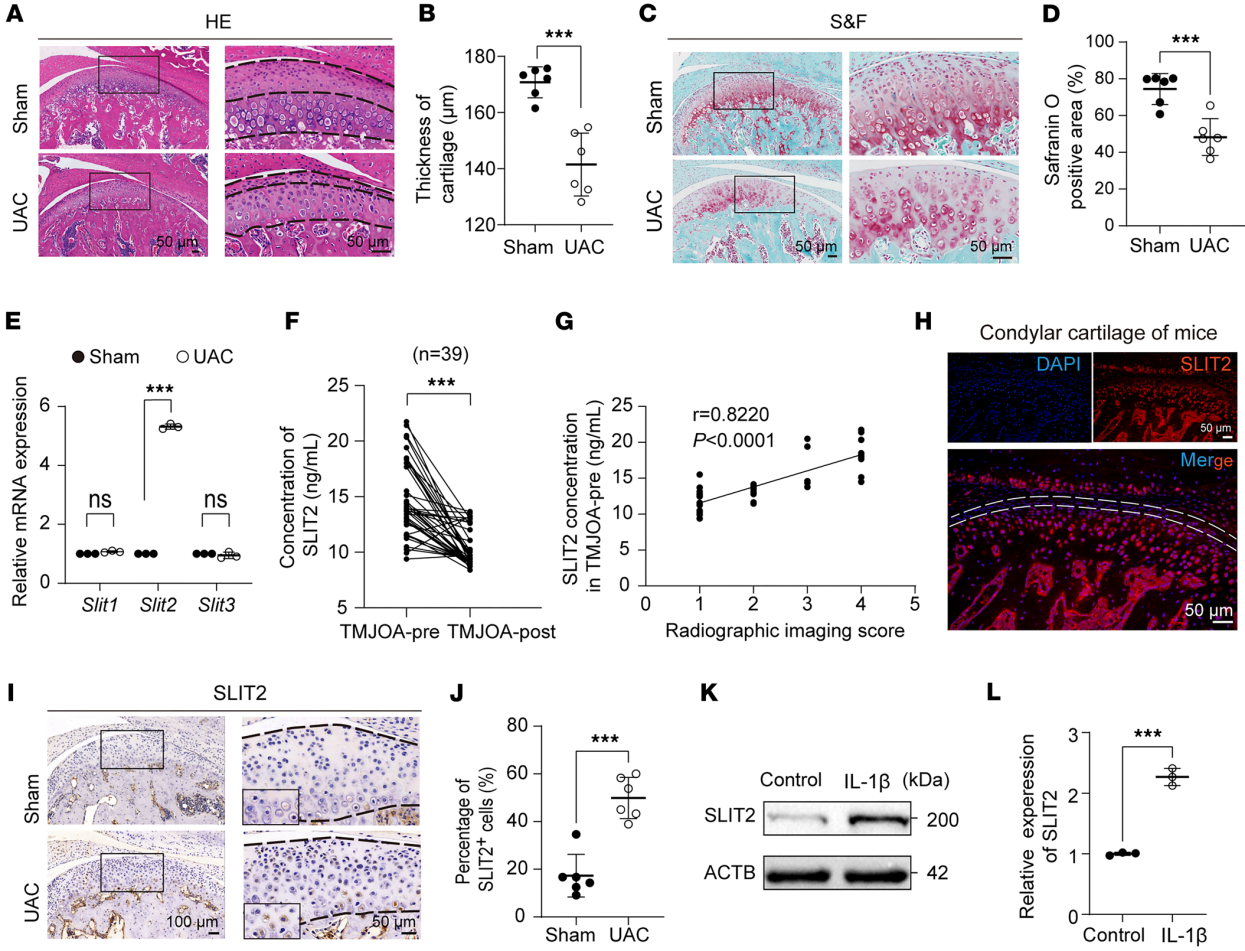
Upregulation of SLIT2 expression in joint tissues in OA. (**A** and **B**) Histological images of condylar cartilage stained with H&E from TMJ tissues of Sham or UAC group, and quantitative analysis of images (*n* = 6). The black dashed lines mark the upper and lower boundaries of the condylar cartilage. (**C** and **D**) Histological images of condylar cartilage stained with S&F from TMJ tissues of Sham or UAC group, and quantitative analysis of images (*n* = 6). (**E**) qRT-PCR analysis of *Slit* family gene expression (*n* = 3) for mouse TMJ tissues, including the disc, articular cartilage, subchondral bone, and synovial tissues. (**F**) ELISA analysis of SLIT2 concentrations in joint lavage fluid from patients with TMJOA before and after treatment (*n* = 39). TMJOA-pre, TMJOA pretreatment; TMJOA-post, TMJOA posttreatment. (**G**) Correlation analysis between radiographic imaging score of TMJOA and the concentration of SLIT2 in TMJOA-pre (*n* = 39). (**H**) IF staining showing SLIT2 localization in the condylar cartilage of 10-week-old mice. (**I** and **J**) Histological images of condylar cartilage stained with IHC for SLIT2 expression from TMJ tissues of Sham or UAC group, and quantitative analysis of images (*n* = 6). (**K** and **L**) Western blotting analysis of SLIT2 expression (*n* = 3). Scale bars: 50 μm (**A**, **C**, **H**, and **I** [right]) and 100 μm (**I** [left]). All data are shown as mean ± SD. Two-tailed Student’s *t* test was used for 2-group comparisons, and Spearman’s rank correlation was applied for correlation analysis. NS, not significant. ***P < 0.001.

Mice subjected to UAC treatment displayed pronounced degenerative changes in condylar cartilage, characterized by thinning of both the superficial and deep zones, a reduction in overall thickness (Figure 1, A and B), and increased matrix degradation (Figure 1, C and D), consistent with prior studies (31–33). In mice with UAC-induced TMJOA, *Slit2* expression was markedly upregulated in the TMJ, while *Slit1* and *Slit3* expression was not (Figure 1E). Enzyme-linked immunosorbent assay (ELISA) analysis of synovial fluid from patients with TMJOA revealed that SLIT2 levels were elevated in the pretreatment group compared with the posttreatment group (Figure 1F). Additionally, SLIT2 levels in synovial fluid were positively correlated with the severity of TMJOA (Figure 1G). Consistently, SLIT2 expression was increased in synovial tissues from TMJOA samples (Supplemental Figure 2, A and B; supplemental material available online with this article; https://doi.org/10.1172/jci.insight.193632DS1). Analysis of the NCBI Gene Expression Omnibus (GEO) single-cell RNA sequencing (scRNA-seq) dataset GSE216651 from knee OA revealed that SLIT2 is primarily expressed in intermediate and lining-layer fibroblasts, with its expression markedly increased under OA conditions (Supplemental Figure 2, C–E). These results offered a possible reason for the elevated levels of SLIT2 in TMJ synovial fluid. We then examined SLIT2 expression in cartilage. Immunofluorescent (IF) staining revealed that SLIT2 was predominantly localized in deep-zone chondrocytes, with minimal expression in the superficial zone (SZ) (Figure 1H), a finding further validated by reanalysis of dataset GSE162823 (Supplemental Figure 2F). In addition, immunohistochemistry (IHC) confirmed higher SLIT2 levels in UAC-induced TMJOA cartilage compared with Sham controls (Figure 1, I and J). Primary chondrocytes were isolated from mouse condylar cartilage (Supplemental Figure 2G) and subsequently treated with IL-1β to mimic inflammatory conditions, which resulted in a marked upregulation of SLIT2 expression (Figure 1, K and L). Human knee OA cartilage samples were collected, and Osteoarthritis Research Society International (OARSI) scoring was performed using safranin O/Fast Green (S&F) staining, as previously described (34). The results demonstrated a positive correlation between SLIT2 levels and the severity of cartilage damage (Supplemental Figure 2, H and I). To identify chondrocyte subpopulations expressing SLIT2 in knee OA, the scRNA-seq dataset GSE255460 was reanalyzed. This analysis revealed that SLIT2 is enriched in fibrocartilage chondrocytes and is upregulated in knee OA cartilage lesions (Supplemental Figure 2, J and K). Additionally, a reanalysis of dataset GSE51588 showed higher SLIT2 expression in subchondral bone associated with damaged cartilage compared with normal tissue (Supplemental Figure 2L).

### SLIT2 overexpression aggravated cartilage degradation in UAC-induced and age-induced TMJOA mice.

**Figure 2:**
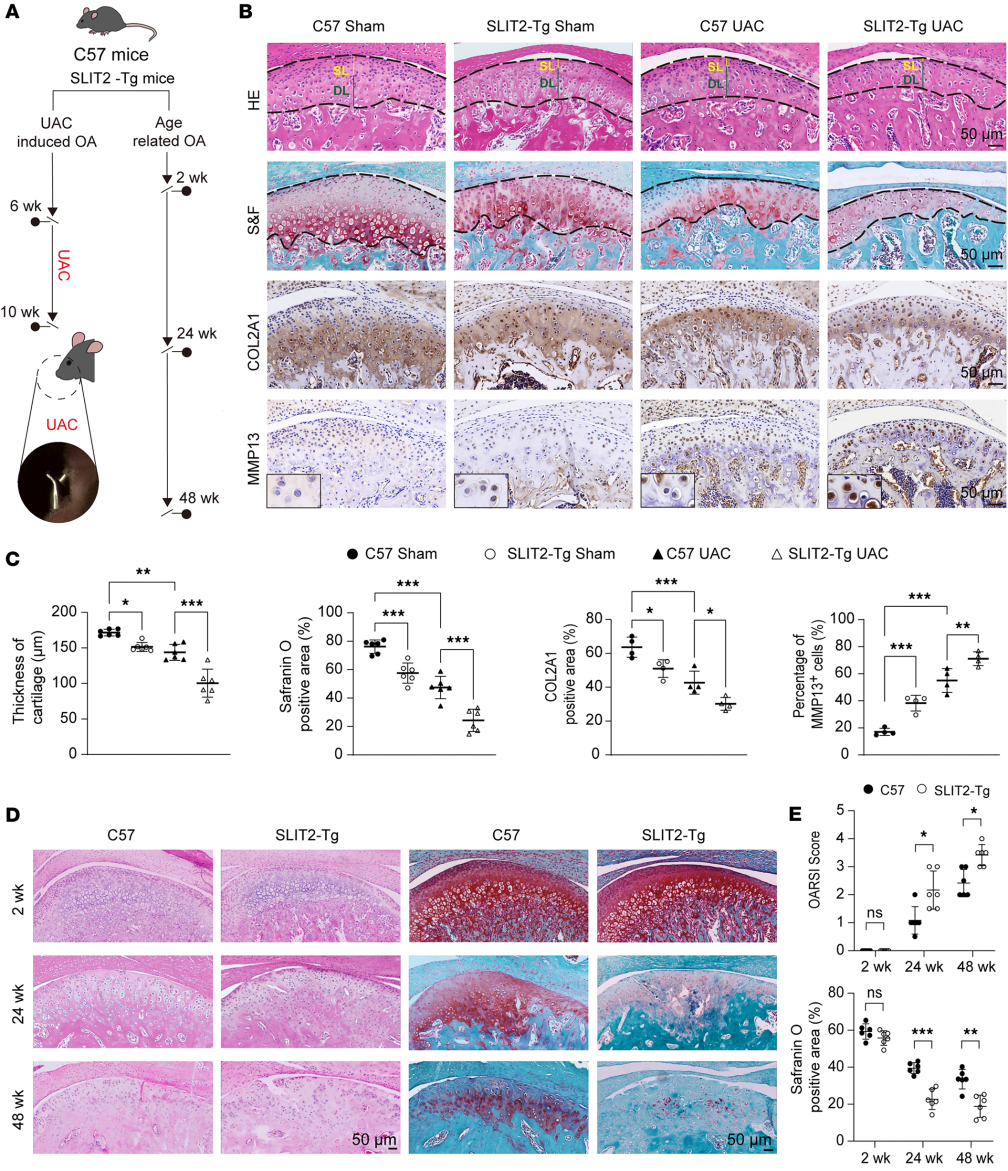
SLIT2 overexpression aggravated cartilage degradation in UAC-induced and age-induced TMJOA mice. (**A**) Flowchart of experimental procedure in vivo. (**B**) Histological images of condylar cartilage stained with H&E, S&F, and IHC for COL2A1 and MMP13. The black dashed lines mark the upper and lower boundaries of the condylar cartilage. The superficial layer (SL) is marked by solid yellow lines. The deep layer (DL) is marked by solid green lines. (**C**) Quantitative analysis of cartilage thickness and S&F-positive area (*n* = 6), and quantitative analysis of COL2A1-positive area (*n* = 4) and percentage of MMP13-positive cells (*n* = 6). (**D**) H&E and S&F staining of 2-week, 24-week, and 48-week condylar cartilage. (**E**) Quantitative analysis of the S&F-positive area and OARSI score (*n* = 6). Scale bars: 50 μm. All data are shown as mean ± SD. Statistical significance was assessed by 2-way ANOVA with Šidák’s post hoc analysis. NS, not significant. *P < 0.05; **P < 0.01; ***P < 0.001.

To investigate the role of SLIT2 in TMJOA progression, *SLIT2*-transgenic (*SLIT2-Tg*) mice with constitutive SLIT2 overexpression (Supplemental Figure 3, A and B) and C57BL/6J (C57) mice as controls were used. Two experimental models were established: UAC-induced TMJOA and age-associated TMJOA (Figure 2A). In the *SLIT2-Tg* Sham group, marked degenerative changes were observed compared with the C57 Sham controls, including a reduction in the thickness of the SZ and proliferative zone, decreased overall cartilage thickness, an increased proportion of the deep zone, a higher prevalence of hypertrophic chondrocytes, and more severe cartilage matrix degradation. Cartilage destruction was more severe in the *SLIT2-Tg* UAC group than in the C57 UAC group. After SLIT2 overexpression, COL2A1-positive areas in the condylar cartilage decreased, while the number of MMP13-positive cells increased, and this trend was more pronounced under UAC induction than in the Sham group (Figure 2, B and C). Meanwhile, the synovium of *SLIT2-Tg* mice exhibited marked thickening and increased inflammatory cell infiltration compared with that of C57 mice. (Supplemental Figure 3, C and D). The incidence of TMJOA increases with age, and aged mice spontaneously develop TMJOA-like pathological changes, which have been observed at 24 and 48 weeks of age (35–38). Consistent with previous reports, our hematoxylin and eosin (H&E) staining revealed that in aging C57 mice, the condylar cartilage surface exhibited fibrosis, irregular chondrocyte organization, acellular regions, and chondrocyte clustering, accompanied by a reduction in S&F-positive cartilage areas. In *SLIT2-Tg* mice, a pronounced loss of proteoglycan staining and the formation of vertical fissures were already evident by 24 weeks of age. By 48 weeks, these mice displayed further surface irregularities, multiple fissures, matrix delamination, marked proteoglycan depletion, and chondrocyte clustering — changes more severe than in C57 mice (Figure 2, D and E). Moreover, MMP13 expression remained higher in *SLIT2-Tg* mice than in C57 mice at 24 and 48 weeks (Supplemental Figure 3, E and F), whereas COL2A1 expression was markedly reduced in *SLIT2-Tg* mice at 24 and 48 weeks (Supplemental Figure 3, G and H).

### SLIT2 overexpression exacerbated subchondral bone loss in UAC-induced TMJOA mice.

**Figure 3:**
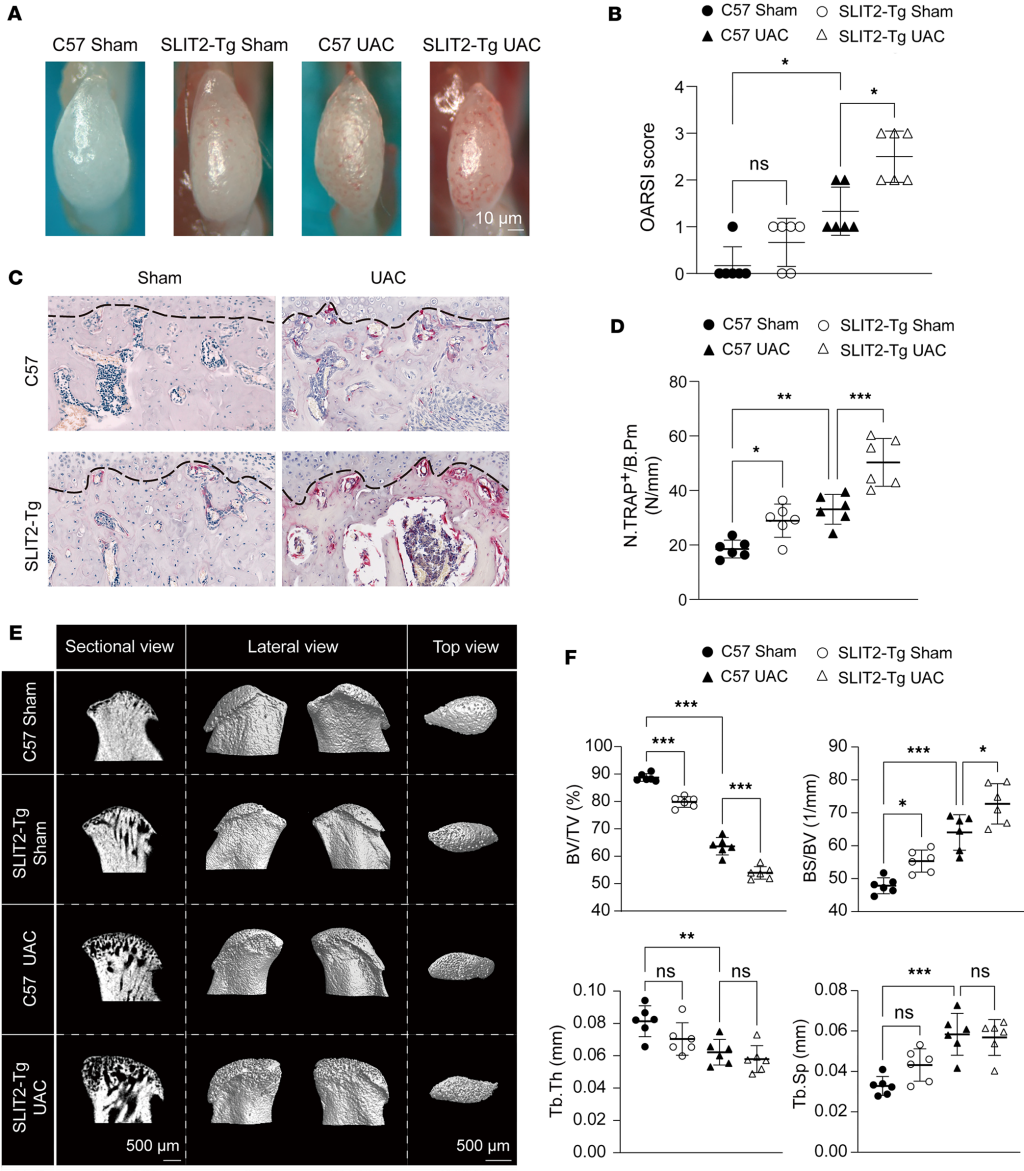
SLIT2 overexpression exacerbated subchondral bone loss in UAC-induced TMJOA mice. (**A** and **B**) Gross appearance of TMJ condyles and morphological scoring based on OARSI criteria (*n* = 6). (**C** and **D**) TRAP staining of the TMJ subchondral bone, and quantitative analysis of TRAP-positive cells per unit of bone perimeter (TRAP^+^/B.pm) (*n* = 6). The black dashed lines denote the upper boundary of the subchondral bone. Scale bar: 50 μm. (**E** and **F**) Two-dimensional and 3-dimensional micro-CT images illustrating the microstructure of subchondral bone, and quantitative analysis using bone structural parameters (*n* = 6). BV/TV, bone volume fraction; BS/BV, bone surface fraction; Tb.Th, trabecular thickness; Tb.Sp, trabecular separation. All data are shown as mean ± SD. Statistical significance was assessed using 2-way ANOVA with Šidák’s post hoc test. NS, not significant. *P < 0.05; **P < 0.01; ***P < 0.001.

Next, we examined TMJOA cartilage morphology and subchondral bone formation after SLIT2 overexpression. Under a medical microscope, normal condylar cartilage surfaces appeared clear and undamaged. After UAC treatment, slight redness appeared on the surface, with more pronounced redness and higher OARSI scores observed in the *SLIT2-Tg* UAC group (Figure 3, A and B). We then performed tartrate-resistant acid phosphatase (TRAP) staining to assess osteoclast activity, which is crucial for evaluating subchondral bone resorption (39). A marked increase in TRAP-positive cells was noted in the subchondral bone of *SLIT2-Tg* mice relative to C57 mice, with a more pronounced elevation observed after UAC induction compared with Sham-operated controls (Figure 3, C and D). Micro-computed tomography (micro-CT) analysis revealed that the bone surface was smooth in the Sham group, whereas UAC induction led to evident surface damage. The extent of bone surface deterioration was more severe in the *SLIT2-Tg* UAC group than in the C57 UAC group (Figure 3E). Quantitative measurements demonstrated markedly lower bone volume fraction (BV/TV) and trabecular thickness (Tb.Th), along with higher bone surface fraction (BS/BV) and trabecular separation (Tb.Sp) in the *SLIT2-Tg* UAC compared with the C57 UAC group (Figure 3F). These findings suggest that transgenic overexpression of *SLIT2* in mice leads to the destruction of subchondral bone, exacerbating the progression of TMJOA.

### SLIT2 led to an inflammatory response and catabolism in chondrocytes.

**Figure 4:**
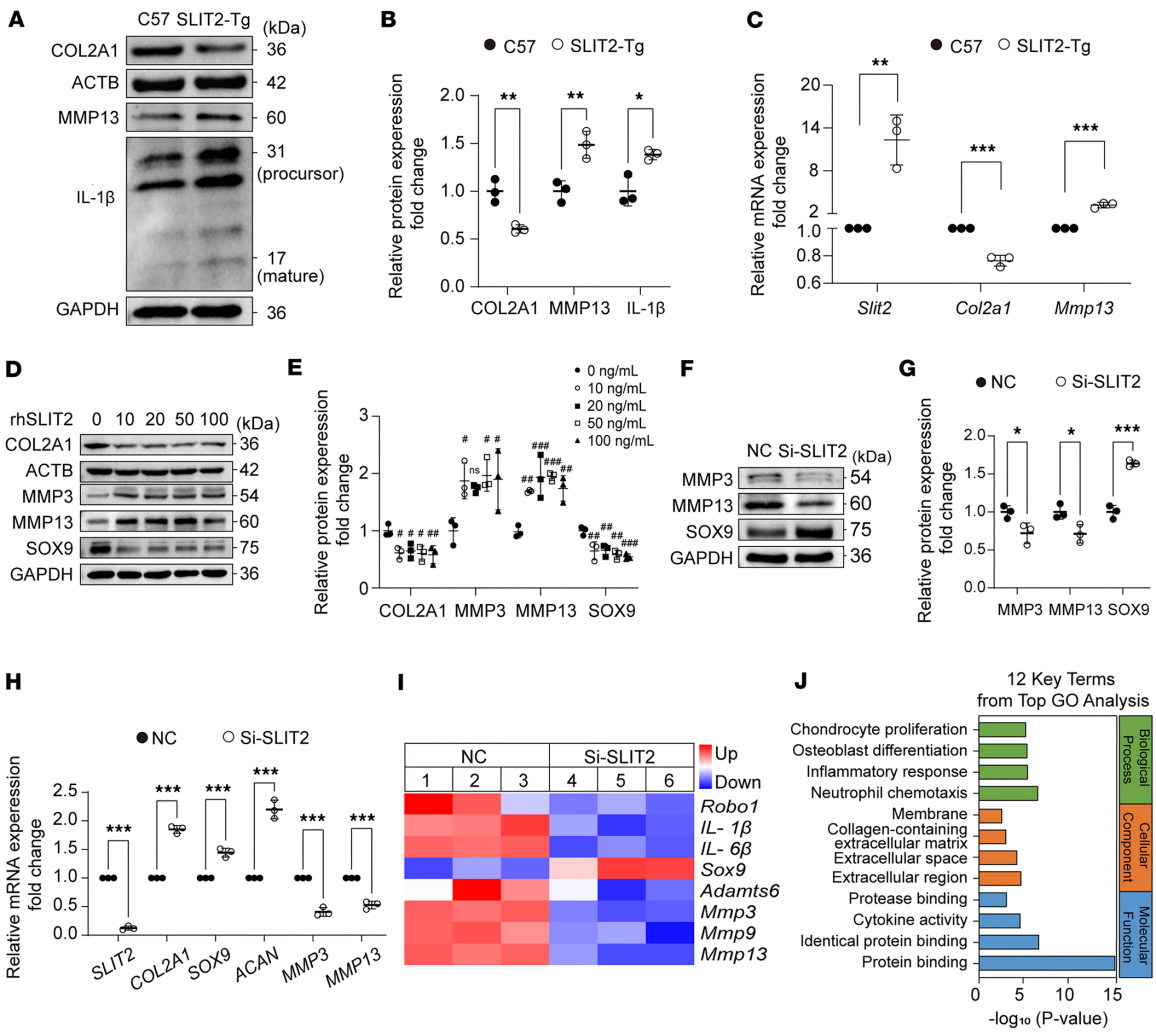
SLIT2 led to an inflammatory response and catabolism in chondrocytes. (**A**) Western blot detection of the effects of SLIT2 overexpression in primary chondrocytes from 3-week-old C57 and *SLIT2-Tg* mice. Primary chondrocytes for each independent isolation batch were pooled from 3 mice, and the process was repeated 3 times. (**B**) Relative quantification of COL2A1, MMP13, and IL-1β proteins. (**C**) qRT-PCR analysis of the effect of SLIT2 overexpression in primary chondrocytes from 3-week-old C57 or *SLIT2-Tg* mice. (**D**) Western blot detection of COL2A1, MMP13, and MMP3 after 48 hours of treatment with rhSLIT2 at 0, 10, 20, 50, and 100 ng/mL in SW1353 cells. (**E**) Relative quantification of COL2A1, MMP3, MMP13 and SOX9 proteins. ^#^*P* < 0.05, ^##^*P* < 0.01, ^###^*P* < 0.001 compared with the 0 ng/mL group. (**F**) Western blot detection of the effect of SLIT2 knockdown on human chondrocytes treated with NC or Si-SLIT2 sequence for 48 hours. (**G**) Relative quantification of MMP3, MMP13, and SOX9 proteins. (**H**) qRT-PCR detection of the effect of SLIT2 knockdown on SW1353 cells treated with NC or Si-SLIT2 sequence for 24 hours. (**I**) A heatmap illustrating differentially expressed genes related to TMJOA from RNA-seq analysis between the NC and Si-SLIT2 groups. (**J**) GO analysis of 12 key terms from the top terms. All data are shown as mean ± SD. Statistical significance was assessed by 2-tailed Student’s *t* test (**B**, **C**, **G**, and **H**) and 1-way ANOVA with Dunnett’s multiple-comparison test (**E**). *P < 0.05; **P < 0.01; ***P < 0.001.

The primary mouse chondrocytes from *SLIT2-Tg* mice exhibited OA-like changes, characterized by reduced COL2A1 protein levels and increased MMP13 protein levels compared with the C57 mice. The expression of the inflammatory factor IL-1β was also upregulated (Figure 4, A and B). Consistent with these protein level changes, *Col2a1* expression was reduced, whereas *Mmp13* expression was increased in SLIT2-overexpressing chondrocytes (Figure 4C). After treating chondrocytes with 10 ng/mL IL-1β, we observed an increase in the expression of *Mmp3* and *Mmp13*, along with a decrease in the expression of *Col2a1* in primary chondrocytes. The knockdown of SLIT2 reversed these effects (Supplemental Figure 4A). We further verified the regulation of SLIT2 expression in chondrocytes in vitro using the human chondrocyte cell line, SW1353. We treated SW1353 cells with 0, 10, 20, 50, and 100 ng/mL recombinant human SLIT2 (rhSLIT2) and performed Western blot analysis after 48 hours. Compared with the 0 ng/mL group, the other treatment groups showed decreased expression of COL2A1 and SOX9 and increased expression of MMP13 and MMP3 (Figure 4, D and E). Knockdown of SLIT2 in chondrocytes decreased the expression of MMP3 and MMP13 and enhanced the expression of SOX9 compared with the negative control (NC) group (Supplemental Figure 4, B and C, and Figure 4, F, and G). qRT-PCR analysis revealed an increase in *ACAN*, *COL2A1*, and *SOX9* expression, and a decrease in *MMP3* and *MMP13* expression (Figure 4H). RNA-seq analysis revealed that markers of inflammation and catabolism in chondrocytes were downregulated following SLIT2 knockdown (Figure 4I). Gene Ontology (GO) analysis highlighted cellular processes related to the inflammatory response, protein binding, and other associated pathways (Figure 4J).

### Knocking down ROBO1 alleviated cartilage degradation in TMJOA.

**Figure 5:**
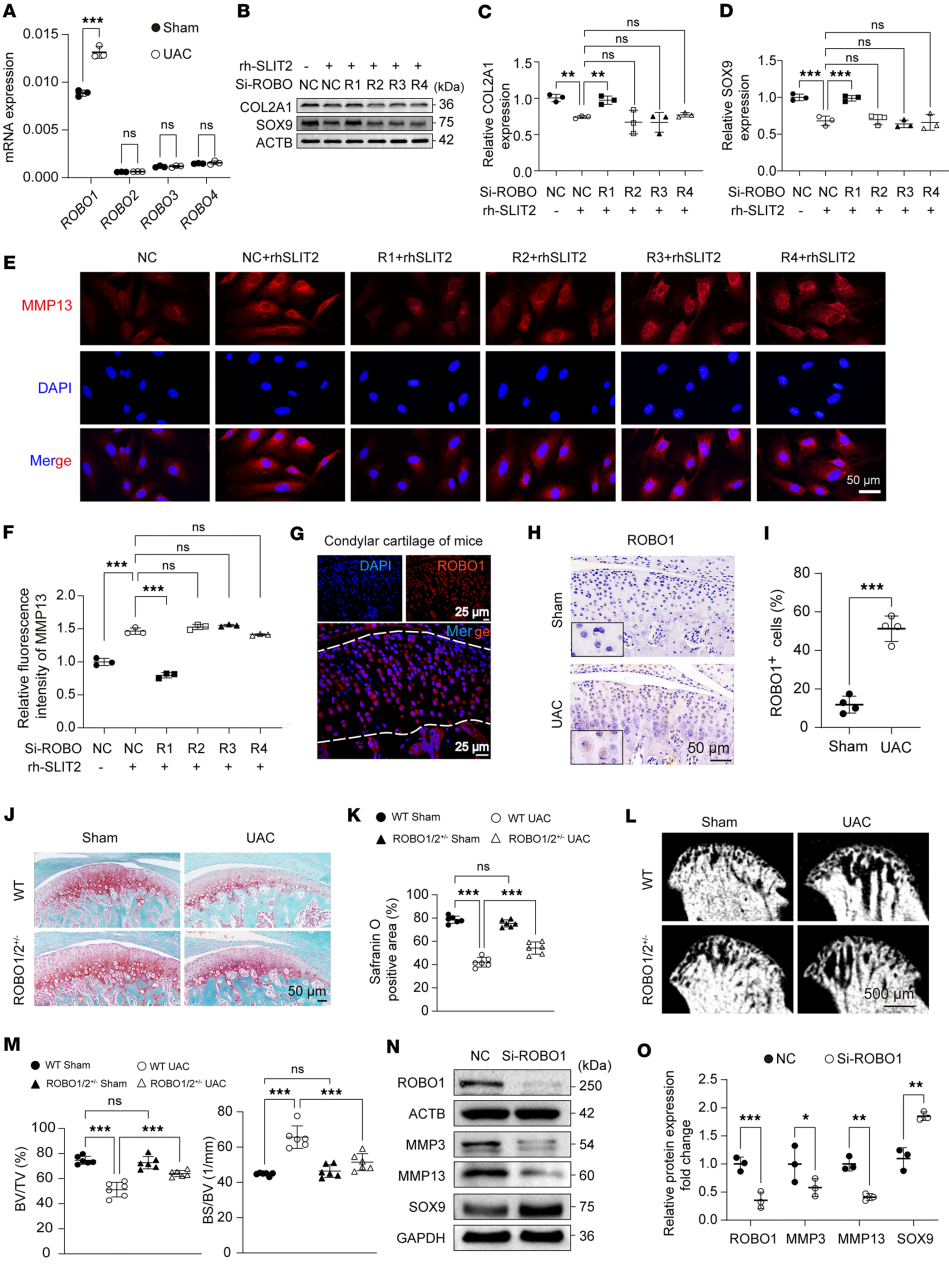
Knocking down ROBO1 alleviated cartilage degradation in TMJOA. (**A**) *Robo* family gene expression levels in cartilage measured by qRT-PCR. (**B**–**D**) Detection of the effect of ROBO1 knockdown in chondrocytes treated with 10 ng/mL rhSLIT2, and relative quantification of the expression of COL2A1 and SOX9 proteins. R1, Si-ROBO1; R2, Si-ROBO2; R3, Si-ROBO3; R4, Si-ROBO4. (**E** and **F**) IF staining showing the effect of ROBO family protein knockdown in chondrocytes treated with 10 ng/mL rhSLIT2, and relative fluorescence intensity of MMP13. (**G**) IF staining showing ROBO1 localization in the condylar cartilage of 10-week-old mice. (**H** and **I**) IHC images of ROBO1 in the condylar cartilage, and quantitative analysis of the percentage of ROBO1-positive cells (*n* = 4). (**J** and **K**) Histological images of condylar cartilage from TMJ tissues stained with S&F, and quantitative analysis of images (*n* = 6). (**L** and **M**) Two-dimensional images illustrating the microstructure of subchondral bone, and quantitative analysis using bone structural parameters (*n* = 6). (**N** and **O**) Detection of the effect of ROBO1 knockdown in chondrocytes, and relative quantification of the expression of ROBO1, MMP3, MMP13, and SOX9 proteins. Scale bars: 50 μm (**E** and **J**), 25 μm (**G**), and 500 μm (**L**). All data are shown as mean ± SD. Statistical significance was assessed by 2-tailed Student’s *t* test (**A**, **I**, and **O**) and 1-way (**C**, **D**, and **F**) or 2-way ANOVA (**K** and **M**) with Šidák post hoc analysis. NS, not significant. *P < 0.05; **P < 0.01; ***P < 0.001.

Having established that SLIT2 accelerates TMJOA progression, we next sought to elucidate the underlying molecular mechanism. Given that the canonical physiological roles of SLIT2, ranging from axon guidance and contact-mediated repulsion to angiogenesis regulation and organ formation, strictly depend on its binding to the ROBO receptor (40, 41), we proceeded to dissect the functional interactions between SLIT2 and the ROBO family in the context of TMJOA pathogenesis. *Robo1* was the most abundantly expressed receptor and exhibited markedly greater upregulation compared with other receptors in the TMJOA cartilage (Figure 5A). Knockdown of ROBO1 reversed the SLIT2-mediated downregulation of COL2A1 and SOX9 protein expression and upregulation of MMP13 in chondrocytes, whereas knockdown of ROBO2–4 receptors had no apparent effect (Figure 5, B–F). ROBO1 was widely expressed across all cartilage layers and various chondrocyte subpopulations (Figure 5G and Supplemental Figure 5A). Notably, ROBO1 expression was upregulated in TMJOA cartilage (Figure 5, H and I) and also showed a pronounced increase in knee OA, mirroring the expression pattern of SLIT2 (Supplemental Figure 5B). These results suggest that SLIT2 primarily promotes cartilage degradation via a ROBO1-mediated mechanism. The regulatory effect of ROBO1 on TMJOA was then further explored in vivo. To circumvent the embryonic lethality associated with complete or partial Robo1 deletion (29, 42), we utilized *Robo1^+/–^Robo2^+/–^* (*Robo1/2^+/–^*) mice, which feature partial deletion of both ROBO1 and ROBO2 receptors, to investigate the role of ROBO1 in TMJOA. Due to the low expression level of ROBO2 in cartilage (Figure 5A and Supplemental Figure 5C) and the fact that its knockdown does not reverse SLIT2-mediated cartilage degradation (Figure 5, B–F), partial ROBO2 deletion has minimal impact on cartilage. Therefore, the *Robo1/2^+/–^* model effectively mimics ROBO1 knockdown in vivo. S&F staining revealed no apparent differences in cartilage matrix between the wild-type (WT) and *Robo1/2^+/–^* groups. However, *Robo1/2^+/–^* mice exhibited a mitigated loss of cartilage matrix induced by UAC compared with WT littermates (Figure 5, J and K). Micro-CT analyses further demonstrated reduced subchondral bone destruction in the *Robo1/2^+/–^* UAC group compared with the WT UAC group (Figure 5L). Specifically, the *Robo1/2^+/–^* UAC group exhibited increased BV/TV and decreased BS/BV relative to the WT UAC group (Figure 5M). The knockdown of ROBO1 in SW1353 cells induced the downregulation of MMP3 and MMP13 expression while upregulating SOX9 expression in vitro (Figure 5, N and O). These results suggest that ROBO1 acts as the main receptor in regulating SLIT2-induced cartilage degradation.

### SLIT2/ROBO1 signaling–induced cartilage degradation was regulated by LRP6-mediated β-catenin signaling.

**Figure 6:**
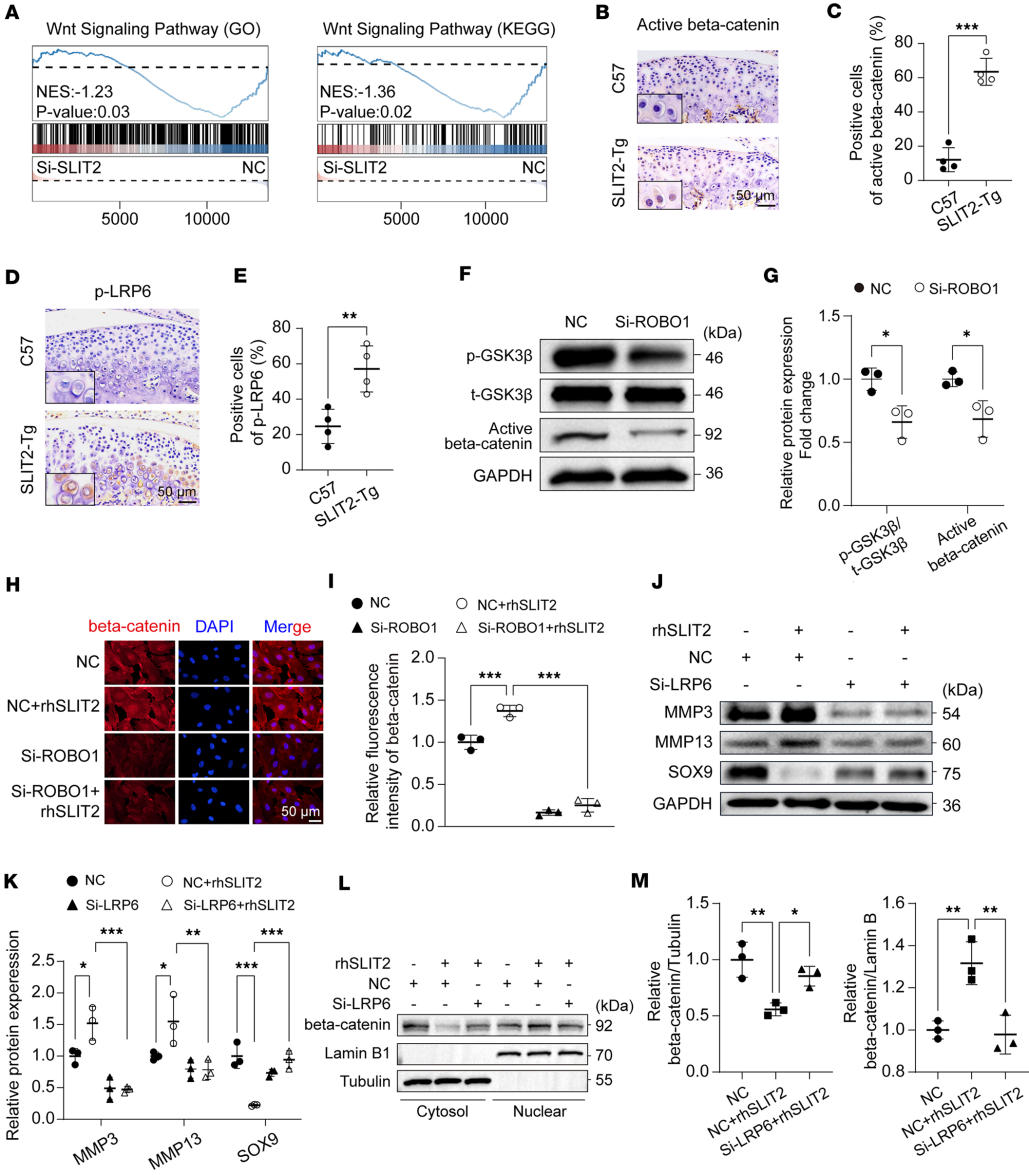
SLIT2/ROBO1 signaling–induced cartilage degradation is regulated by LRP6-mediated β-catenin signaling. (**A**) GSEA was performed using gene sets related to the Wnt/β-catenin pathway from the GO and KEGG databases to assess differences between the NC and Si-SLIT2 groups. (**B** and **C**) IHC images of active β-catenin in condylar cartilage, and quantitative analysis of the percentage of active β-catenin–positive cells (*n* = 4). (**D** and **E**) IHC images of p-LRP6 in condylar cartilage, and quantitative analysis of the percentage of p-LRP6–positive cells (*n* = 4). (**F** and **G**) Western blot detection of the effect of ROBO1 knockdown for GSK-3β and active β-catenin proteins in SW1353 chondrocytes, and relative quantification of p-GSK-3β/t-GSK-3β and active β-catenin proteins. (**H** and **I**) IF staining images of β-catenin proteins after being treated with rhSLIT2 and/or Si-ROBO1, and relative quantification of fluorescence intensity of β-catenin proteins. (**J** and **K**) Western blot analysis assessing the effect of LRP6 knockdown on rhSLIT2-induced catabolism in SW1353 chondrocytes, and relative quantification of MMP3, MMP13, and SOX9 proteins. (**L** and **M**) Western blot analysis showing the impact of LRP6 knockdown on rhSLIT2-induced nuclear translocation of β-catenin in SW1353 cells, and relative quantification of β-catenin proteins in the cytoplasm and nucleus. Scale bars: 50 μm. All data are shown as mean ± SD. Statistical significance was assessed by 2-tailed Student’s *t* test (**C**, **E**, and **G**), 2-way ANOVA with Šidák’s post hoc analysis (**I** and **K**), and 1-way ANOVA with Dunnett’s multiple-comparison test (**M**). *P < 0.05; **P < 0.01; ***P < 0.001.

Gene set enrichment analysis (GSEA) revealed suppression of Wnt/β-catenin signaling following SLIT2 knockdown (Figure 6A). IHC in *SLIT2-Tg* mice showed increased nuclear translocation of active β-catenin in chondrocytes (Figure 6, B and C), along with elevated levels of phosphorylated LRP6 (p-LRP6) (Figure 6, D and E). Phosphorylated GSK-3β (p-GSK-3β) represents an inactive state that reduces the ability to degrade β-catenin (43). Following ROBO1 knockdown, total GSK-3β (t-GSK-3β) protein levels remained unchanged; however, its phosphorylation was markedly reduced, concomitant with a decrease in active β-catenin levels (Figure 6, F and G). IF staining further showed that treatment with rhSLIT2 increased β-catenin expression and enhanced its nuclear translocation, effects that were reversed by ROBO1 knockdown in chondrocytes (Figure 6, H and I). LRP6 is a key upstream receptor of β-catenin (44). The rhSLIT2-induced increase in MMP3 and MMP13 and the decrease in SOX9 were reversed upon LRP6 knockdown (Figure 6, J and K, and Supplemental Figure 5D). Treatment with rhSLIT2 promoted nuclear translocation of β-catenin; however, this effect was reversed by LRP6 knockdown (Figure 6, L and M). These results suggest that SLIT2/ROBO1 signaling–induced cartilage degradation is regulated by LRP6-mediated β-catenin signaling.

### SLIT2 enhanced ROBO1-LRP6 complex formation and LRP6 phosphorylation.

**Figure 7:**
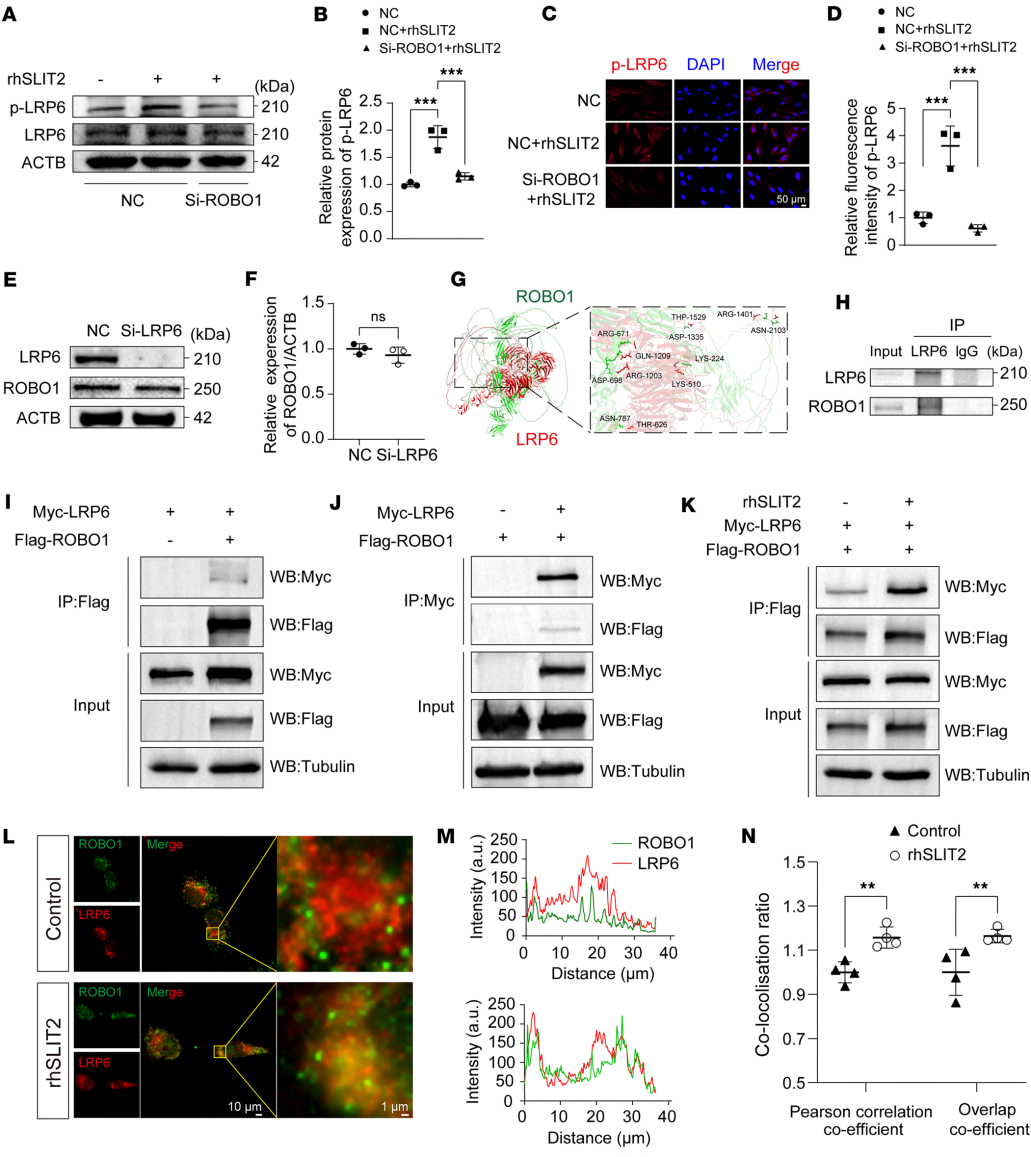
SLIT2 enhanced ROBO1-LRP6 complex formation and LRP6 phosphorylation. (**A** and **B**) Western blot analysis of p-LRP6 with the effect of rhSLIT2 and/or Si-ROBO1 in SW1353 cells, and relative quantification of p-LRP6 proteins. (**C** and **D**) IF staining images of the effect of rhSLIT2 and Si-ROBO1 on the expression of p-LRP6 in SW1353 cells, and relative quantification of fluorescence intensity of p-LRP6 proteins. Scale bar: 50 μm. (**E** and **F**) Western blot analysis examining the effect of LRP6 knockdown on ROBO1 expression in SW1353 cells, and relative quantification of ROBO1 proteins. (**G**) Visualization of representative potential binding sites between LRP6 and ROBO1 using PyMOL. (**H**) Co-IP experiments detected the interaction between ROBO1 and LRP6 in SW1353 cells. (**I** and **J**) Co-IP experiments using overexpression plasmids for LRP6 and ROBO1 to validate their interaction in SW1353 chondrocytes. (**K**) Co-IP analysis of the effect of rhSLIT2 on the interaction between LRP6 and ROBO1 in SW1353 chondrocytes transfected with overexpression plasmids. (**L**) IF staining showing the effect of rhSLIT2 on the colocalization of ROBO1 and LRP6 in SW1353 cells. Scale bars: 10 μm (left) and 1 μm (right). (**M**) IF staining intensity curve of ROBO1 and LRP6 in IF images. (**N**) Quantification of colocalization using Pearson’s correlation and overlap coefficients. All data are shown as mean ± SD. Statistical significance was assessed by 1-way ANOVA with Dunnett’s multiple-comparison test (**B** and **D**) and 2-tailed Student’s *t* test (**F** and **N**). NS, not significant. **P < 0.01; ***P < 0.001.

We next examined the relationship between SLIT2/ROBO1 and LRP6-associated β-catenin signaling. Western blot analysis indicated that rhSLIT2 enhanced LRP6 phosphorylation, an effect reversed by ROBO1 knockdown (Figure 7, A and B). IF staining also showed that rhSLIT2 increased the fluorescence intensity of p-LRP6; however, this increase was attenuated by ROBO1 knockdown (Figure 7, C and D). ROBO1 expression remained unchanged following LRP6 knockdown (Figure 7, E and F). These results indicate that SLIT2/ROBO1 promotes LRP6 phosphorylation, thereby activating β-catenin signaling. SLIT2 binding induces a conformational change in ROBO1, leading to the recruitment of kinases and activation of downstream signaling pathways (9, 45). Similarly, Wnt binding to Frizzled induces a conformational change that promotes the interaction between Frizzled and LRP6 and recruits intracellular signaling proteins, thereby facilitating LRP6 phosphorylation (46, 47). Based on these parallels, we hypothesized that ROBO1 and LRP6 may form a complex through a mechanism analogous to that of the Frizzled-LRP6 interaction. To explore this possibility, we analyzed the GEPIA platform database, which revealed a positive correlation between ROBO1 and LRP6 expression (Supplemental Figure 6A). Bioinformatics analysis showed the binding free energy of ROBO1-LRP6 complex was –322.69 kcal/mol (Supplemental Table 5), with multiple amino acid clusters identified at both extracellular and intracellular binding sites (Figure 7G). To validate these findings experimentally, we performed co-IP assays in SW1353 chondrocytes. ROBO1 was detected in the LRP6-immunoprecipitated complex (Figure 7H). Furthermore, SW1353 chondrocytes were transfected with FLAG-tagged ROBO1 or Myc-tagged LRP6 expression constructs (Supplemental Figure 6B). Co-IP assays revealed that Myc-tagged LRP6 was detected in FLAG-tagged ROBO1 immunoprecipitates isolated using anti-FLAG nanobody agarose beads (Figure 7I). Reciprocally, FLAG-tagged ROBO1 was detected in complexes pulled down with Myc-tagged LRP6 (Figure 7J). Following rhSLIT2 treatment, co-IP analysis revealed increased enrichment of FLAG-tagged ROBO1 in Myc-tagged LRP6 immunoprecipitates (Figure 7K). Consistently, IF staining demonstrated that treatment with rhSLIT2 enhanced the colocalization of ROBO1 and LRP6 in chondrocytes (Figure 7, L–N).

## Discussion

This study examined the role of the SLIT2/ROBO1/LRP6 axis in TMJOA progression. Initially, we identified elevated SLIT2 levels in the synovial fluid of patients with TMJOA and the TMJ tissues of UAC mice. Subsequently, using *SLIT2-Tg* and *Robo1/2^+/–^* mice, we demonstrated that the activation of SLIT2/ROBO1 signaling contributed to the progression of TMJOA. Furthermore, in vivo and in vitro experiments showed that SLIT2 promotes ROBO1-LRP6 complex formation, thereby activating the β-catenin pathway and facilitating TMJOA progression.

In this study, we observed that SLIT2 was predominantly localized in the deep zone of cartilage, with much lower expression in the SZ. This spatial distribution pattern is consistent with that of classical Wnt/β-catenin signaling molecules, particularly β-catenin that is known to promote chondrocyte hypertrophy, calcification, and OA progression (48). The SZ harbors fibrochondrocyte stem cells (FCSCs), which function as progenitor cells essential for cartilage repair and maintenance. Previous studies have shown that excessive activation of the Wnt/β-catenin signaling pathway leads to the depletion of FCSCs, whereas the maintenance of FCSCs in the SZ requires a microenvironment characterized by low Wnt/β-catenin activity (49). Therefore, the low SLIT2 expression in the SZ likely reflects a physiological state that preserves this low–β-catenin–signaling environment essential for SZ homeostasis. In contrast, SLIT2 overexpression in our model led to enhanced β-catenin expression, accompanied by SZ thinning, thickening of the mature and hypertrophic cell layers, cluster-like cellular aggregation, and TMJOA-like pathological changes. These findings suggest that SLIT2 spatial distribution supports a positive regulatory role in chondrocyte terminal differentiation and TMJOA progression.

Our analysis of clinical TMJOA samples and in vivo experiments revealed an increase in SLIT2 and ROBO1 expression, which was further validated by reanalysis of the scRNA-seq data. The upregulation of SLIT2 and ROBO1 has also been reported in other chronic low-grade inflammatory diseases, including systemic lupus erythematosus, gestational diabetes, and Alzheimer disease (14, 16, 50, 51), suggesting a potentially broad association between SLIT2/ROBO1 signaling and inflammation. Subsequent experiments revealed that SLIT2 knockdown suppressed chondrocyte inflammatory responses, mirroring previous reports where SLIT2-ROBO1/2 blockade attenuated ocular neovascular inflammation (41). Collectively, these findings demonstrate that the SLIT2/ROBO1 signaling axis is activated in TMJOA and regulates disease progression.

To further validate the role of SLIT2 and ROBO1 signaling in vivo, we utilized *SLIT2-Tg* mice and *Robo1/2^+/–^* mice. Our results revealed that SLIT2/ROBO1 activation reduced the cartilage thickness and promoted matrix degradation in UAC-induced TMJOA. Mechanistically, SLIT2 upregulated the cartilage-degrading enzyme MMP13, whereas knockdown of ROBO1, but not ROBO2, ROBO3, or ROBO4, reversed its expression. This finding is consistent with previous reports showing that SLIT2/ROBO1 signaling suppresses TIMP expression (52–54), thereby enhancing MMP activity. Additionally, the SLIT2/ROBO1 signaling has been shown to be enhanced by heparan sulfate (HS) sulfation, which is elevated in OA (9, 55, 56). This suggests that the cartilage-destructive effects of SLIT2/ROBO1 signaling may be further amplified in OA owing to increased HS sulfation. In addition to cartilage degradation in UAC-induced TMJOA, *SLIT2-Tg* mice exhibited increased osteoclast numbers and subchondral bone resorption, whereas these bone changes were attenuated in *Robo1/2^+/–^* mice. According to previous studies, SLIT2 overexpression aggravated bone resorption by promoting osteoclastogenesis and suppressing osteoblast differentiation (17, 18). The bone defects observed in UAC or aging *SLIT2-Tg* mice likely arise from both enhanced cartilage destruction and an impaired subchondral bone turnover process, highlighting the multifaceted role of SLIT2 in TMJOA pathogenesis. Future studies employing conditional knockout models to precisely research the temporal and spatial functions of SLIT2 in both bone and cartilage are essential for explaining the complex interplay governing TMJOA pathogenesis.

The Wnt/β-catenin pathway regulates complex cellular functioning during embryonic development and pathogenesis of diseases. The dysregulation of β-catenin can induce cartilage destruction and an OA-like phenotype in transgenic mice, suggesting that moderate β-catenin activity is necessary for the physiological maintenance of cartilage (57). Our data demonstrated that rhSLIT2 promoted β-catenin activation in chondrocytes, an effect reversed by ROBO1 knockdown. Previous studies have indicated that activating the β-catenin signaling in condylar chondrocytes results in cartilage degeneration and subchondral bone erosion (48, 58), further supporting our findings. Several studies indicated that the SLIT2/ROBO1 signaling can block β-catenin signaling via the TGF-β/GSK-3β or PI3K/AKT pathway, suppressing tumorigenesis (59–61). Therefore, we can deduce that SLIT2/ROBO1 signaling activates β-catenin signaling in a different manner in chondrocytes from that in cancer cells. In the context of chondrocytes, our study suggests that SLIT2/ROBO1 mediates β-catenin nuclear translocation in an LRP6-dependent manner. These results not only advance our understanding of SLIT2/ROBO1 signaling plasticity across cellular contexts but also provide critical insights into the pathogenesis of TMJOA.

Our work supports crosstalk between SLIT2 and Wnt/β-catenin pathways mediated by ROBO1-LRP6 interaction. We found that SLIT2 enhanced LRP6 phosphorylation, an effect that was reversed upon ROBO1 knockdown, consistent with prior observations of the ability of SLIT2/ROBO1 signaling to induce Src phosphorylation (24). We further discovered that ROBO1 interacted with LRP6, and the molecular docking results suggested potential binding sites inside and outside the cell membrane. Remarkably, our results further demonstrated that rhSLIT2 treatment strengthened the interaction between ROBO1 and LRP6. This ligand-induced receptor clustering mirrors mechanisms observed in neural development, where SLIT2 facilitates ROBO1-Dscam1 coreceptor complex formation to promote axonal growth (9, 62). These parallels suggest that SLIT-mediated receptor assembly is a mechanism conserved across biological systems.

This study has several notable limitations. Although the regulatory effects of SLIT2 on bone turnover have been reported previously, the specific mechanisms by which SLIT2 promote subchondral bone loss in TMJOA need thorough investigation. Furthermore, the ROBO-LRP6 interaction has not been fully characterized, and future studies are needed to clarify the precise binding sites and the biological functioning associated with this interaction. While silencing ROBO1 has shown efficacy in mitigating TMJOA-related cartilage degradation, its clinical application may face challenges. In contrast, the R5 monoclonal antibody, which specifically neutralizes the SLIT2-ROBO1 interaction by targeting the first immunoglobulin-like domain of ROBO1 (42, 52), offers a more feasible and promising therapeutic strategy.

In conclusion, our study showed that activation of the SLIT2/ROBO1/LRP6 axis promotes inflammation and cartilage degradation via β-catenin signaling in TMJOA. This mechanism may provide insights into potential therapeutic strategies for treating TMJOA in clinical settings.

## Methods

### Sex as a biological variable.

Female mice were chosen for this study to align with clinical evidence indicating a higher prevalence of TMJOA in female patients compared with males (63, 64).

### Human TMJ lavage fluid and knee OA samples.

Both pre- and posttreatment TMJ lavage fluid samples were collected from 39 patients with clinically diagnosed TMJOA who underwent hyaluronic acid therapy at the Affiliated Stomatology Hospital of Guangzhou Medical University, Guangzhou, China. Pretreatment samples were collected at the initial assessment, where TMJOA was diagnosed based on clinical symptoms and cone-beam CT (CBCT) findings. Synovial fluid was aspirated, and baseline parameters (maximal mouth opening, Numeric Rating Scale [NRS] pain score, and imaging score) were recorded. Posttreatment samples were obtained after completion of hyaluronic acid therapy, along with reassessment of mouth opening and NRS pain score (65). Knee OA samples were obtained from 5 patients who required joint replacement surgeries at the Guangdong Provincial People’s Hospital, Guangzhou, China. All the patients provided informed consent to participate in the study. Clinical information about the patients is provided in Supplemental Tables 1 and 2.

### Animals.

*Robo1/2^+/–^* mice, their WT littermates, and *SLIT2-Tg* mice were obtained from Lijing Wang’s lab, School of Life Sciences and Biopharmaceutics, Guangdong Pharmaceutical University (Guangzhou, China). C57 mice were obtained from the Guangdong Medical Laboratory Animal Center (Guangzhou, China). Detailed animal generation, breeding, and PCR genotyping procedures have been described in previous studies (42, 66, 67). Mice were randomly divided into Sham and UAC groups. For the UAC model, 6-week-old mice were anesthetized, and a properly sized metal tube was affixed to the left maxillary and mandibular incisors using an adhesive. The metal tube on the mandibular incisor was curved to 135° to form a cross-bite relationship with the metal tube on the maxillary incisor (Supplemental Figure 1A). The metal tubes were inspected regularly to ensure integrity. The identical surgical procedure was conducted on the Sham group, but without the attachment of metal tubes.

### Data collection and preprocessing.

Three publicly accessible scRNA-seq datasets (GSE216651, GSE255460, and GSE104782) were analyzed following the methodologies described in the original publications associated with each dataset (68–70). The analysis was performed using the Seurat package (version 4.2.1) in R software (https://satijalab.org/seurat/). The standard Seurat pipeline was used for data preprocessing and analysis. The initial quality control filtered out low-quality or unreliable cells. Data were normalized using the normalized data function. A Uniform Manifold Approximation and Projection achieved dimensionality reduction. Clustering was performed using the Louvain algorithm at a resolution of 0.8. Pseudotime trajectory analysis was conducted using the Slingshot package to explore developmental or differentiation processes across the identified clusters. Expression of the genes of interest (SLIT1–3 and ROBO1–4) was visualized using feature plots and dot plots within Seurat. Two publicly available bulk RNA-seq datasets (GSE162823 and GSE51588) were analyzed using the DESeq2 R package (https://bioconductor.org/packages/DESeq2/). Data were filtered for low-expression genes and normalized using size-factor normalization. For the dataset GSE162823, gene expression levels were visualized using heatmaps. For the GSE51588 dataset, differentially expressed genes were identified based on the criteria of |log_2_(fold change)| greater than 1.5 and *P* value less than 0.05 and visualized using volcano plots. The GEPIA database was used to predict the correlation between ROBO1 and LRP6 (http://gepia.cancer-pku.cn/).

### ELISA.

Human TMJ lavage fluid samples were centrifuged at 3,000*g* and 4°C to obtain supernatants. The concentration of SLIT2 in the supernatant was quantified using an ELISA kit (SEA672Hu, Cloud-Clone Corporation).

### Representative volumetric microscopic images.

Condyles were imaged using a medical microscope (Thermo Fisher Scientific). Surface morphology was assessed and scored using the OARSI standard scoring system, as detailed in previous studies (65).

### Histological analysis.

Tissues were preserved in 4% paraformaldehyde (PFA) and subjected to decalcification using EDTA. Paraffin-embedded specimens were sectioned to a thickness of 4 μm and stained with H&E (G1076, Servicebio) or S&F (G1053, Servicebio). Osteoclasts were identified using TRAP staining (G1050, Servicebio).

### Micro-CT.

The mouse heads were fixed in 4% PFA for imaging using a Skyscan 1173 system (Bruker). Scanning parameters included an acceleration voltage of 59 kV and a beam current of 100 μA with a resolution of 9.0 μm.

### IHC.

Mouse TMJ tissue was preserved in 4% PFA for 24 hours and decalcified in 10% EDTA. Paraffin-embedded specimens were sectioned at 4 μm thickness. The sections were deparaffinized, rehydrated, and treated for antigen retrieval. Endogenous peroxidase activity was inhibited with 3% H_2_O_2_. Sections were blocked with blocking buffer (P0260, Beyotime) and incubated overnight at 4°C with primary antibodies against SLIT2 (1:100; ab134166, Abcam), ROBO1 (1:200; 67922-1-Ig, Proteintech), COL2A1 (1:100; BA0533, Boster), MMP13 (1:100; ab39012, Abcam), active β-catenin (Ser33/37/Thr41) (1:100; 8814, Cell Signaling Technology), and p-LRP6 (Ser1490) (1:100; P42570-1, Abmart). HRP-conjugated goat anti-rabbit IgG (L3012) and goat anti-mouse IgG (L3032) secondary antibodies were applied and visualized with DAB. Counterstaining was performed using hematoxylin. The sections were then dehydrated, cleared with xylene, and mounted.

### Primary chondrocytes and human chondrocyte cell lines.

Primary chondrocytes were obtained from 3-week-old animals’ condylar cartilage. Each independent batch of primary chondrocyte isolation was performed using cells pooled from 3 mice, and this procedure was repeated 3 times independently to ensure biological reproducibility. First, cartilage samples were digested with 0.25% trypsin (Gibco) at 37°C for 30 minutes, then with 0.2% collagenase II (Sigma-Aldrich) for 4 hours. Primary cells were validated by cell morphology, toluidine blue staining, and type II collagen IF staining. HEK293T cells and the human chondrocyte cell line SW1353 were purchased from Procell Life Science & Technology Co., Ltd. and cultured in DMEM containing 10% fetal bovine serum (FBS) and 1% penicillin/streptomycin (P/S). rhSLIT2 (R&D Systems) or IL-1β (PeproTech) was added when the cells reached the appropriate density.

### RNA-seq.

RNA-seq of chondrocytes with NC or Si-SLIT2 treatment was performed. The total RNA was extracted and assessed for quality. The mRNA was purified, fragmented, and used for cDNA synthesis. After adapter ligation and fragment selection, the libraries were enriched via PCR and sequenced on an Illumina NovaSeq 6000 platform by Shanghai Personal Biotechnology Co., Ltd. The DESeq2 R package was used for differential expression analysis. Differentially expressed genes between the NC and Si-SLIT2 groups were identified using the criteria of |log_2_(fold change)| greater than 1.5 and a *P* value of less than 0.05. GO, Kyoto Encyclopedia of Genes and Genomes (KEGG) enrichment, and GSEA analyses were conducted using the ClusterProfiler R package (https://bioconductor.org/packages/clusterProfiler/).

### IF staining.

Chondrocytes were seeded onto 24-mm cell slides (J06001, Jing An Bio) in 6-well plates (CCP-6H, Servicebio). Cells were fixed with 4% PFA and permeabilized with Triton X-100 (P0013B, Beyotime). After treating with blocking buffer (P0260, Beyotime) for 30 minutes, cells were incubated overnight at 4°C with primary antibodies against β-catenin (Ser33/37/Thr41) (1:200; 8814, Cell Signaling Technology), ROBO1 (1:200; 67922-1-Ig, Proteintech), p-LRP6 (1:100; P42570-1, Abmart), and LRP6 (1:200; T58345, Abmart). After washing, cells were incubated for 1 hour at 37°C in the dark with Alexa Fluor 594–conjugated donkey anti-rabbit IgG (1:200; 34212ES60, Yeasen Biotechnology) and/or Alexa Fluor 488–conjugated donkey anti-mouse IgG (1:200; 34106ES60, Yeasen Biotechnology). Fluorescence images were acquired via an inverted fluorescence microscope.

### siRNA and cell transfection.

Chondrocytes were transiently transfected with siRNA in Opti-MEM (31985070, Invitrogen) using GP-transfect-Mate (G04008, GenePharma) following the manufacturer’s protocol. After 48 hours of incubation, the cells were treated accordingly. siRNA was synthesized by GenePharma, and the sequences used are listed in Supplemental Table 3.

### qRT-PCR.

According to the manufacturer’s instructions, the Tissue RNA Purification Kit Plus (EZB-RN001-plus, EZBioscience) and Express RNA Purification Kit (B0004DP, EZBioscience) were used to extract tissue RNA and cellular RNA. NanoDrop 2000 spectrophotometers (Thermo Fisher Scientific) were used to assess RNA concentration. For reverse transcription, the Color Reverse Transcriptase Kit (EZBioscience) was used. Gene expression was analyzed by qRT-PCR using SYBR Green qPCR Mix (A0012-R2, EZBioscience). Specific primers for human and mouse genes are listed in Supplemental Table 4. The expression levels were standardized to those of GAPDH.

### Western blot analysis.

RIPA Lysis Buffer (P0013B, Beyotime) was used to extract equal amounts of protein from tissues and cells. The Nuclear Protein Extraction Kit (P0028, Beyotime) was used to separate nuclear/cytoplasmic proteins. Proteins were separated by SDS-PAGE and transferred to PVDF membranes (IPVH00010, Millipore). One hour after blocking with 5% nonfat powdered milk (P0216, Beyotime) at room temperature, the membrane was incubated with primary antibodies targeting SLIT2 (1:1000; ab134166, Abcam), IL-1β (1:1000; 26048-1-AP, Proteintech), COL2A1 (1:1000; ab188570, Abcam), MMP13 (1:1000; ab39012, Abcam), MMP3 (1:1000; ab52915, Abcam), SOX9 (1:1000; ab185966, Abcam), ROBO1 (1:3000; 67922-1-Ig, Proteintech), GSK-3β (1:5000; 22104-1-AP, Proteintech), p-GSK-3β (1:1000; 5558, Cell Signaling Technology), active β-catenin (Ser33/37/Thr41) (1:1000; 8814, Cell Signaling Technology); p-LRP6 (1:100; P42570-1, Abmart), LRP6 (1:1000; 3395, Cell Signaling Technology), GAPDH (1:20,000; 60004-1-Ig, Proteintech), β-actin (1:20,000; 66009-1-Ig, Proteintech), lamin B1 (1:5000; 12987-1-AP, Proteintech), and α-tubulin (1:10,000; 11224-1-AP, Proteintech). The cells were then incubated with HRP-conjugated goat anti-rabbit/mouse IgG (H+L) (1:10,000; L3012/L3032, Signalway Antibody) for 1 hour. Detection was performed using a chemiluminescent detection substrate (WBKLS, Millipore).

### Molecular docking screening.

The docking experiments employed ROBO1 (UniProt: Q9Y6N7) and LRP6 (UniProt: O75581) as target proteins. Protein-protein interaction models and docking simulations were conducted using AlphaFold3 (71). The model with the highest-ranking score was identified to have the optimal binding configuration. To assess the free binding energy of the selected model, the HawkDOCK SERVER (http://cadd.zju.edu.cn/hawkdock/) was used to rank the binding energies of individual amino acid residues and evaluate their respective contributions to the interactions (72, 73) (Supplemental Table 5). Lastly, the binding sites with the most substantial contributions were visualized using PyMOL 2.4 software (https://pymol.org/), facilitating an intuitive analysis of the protein-protein interactions.

### Co-IP.

Overexpression plasmids for ROBO1 tagged with FLAG and LRP6 tagged with Myc were synthesized (OBiO Technology). The cells were plated in 100-mm dishes. Transfection mixtures were prepared using Opti-MEM medium, plasmid, and EZ Trans cell transfection reagent (AC04L091, LifeiLab) and then applied to the cells. IP was performed using Anti-FLAG Nanobody Agarose Beads (KTSM1338, Alpalifebio) and Anti-Myc Nanobody Agarose Beads (KTSM1336, Alpalifebio). Western blotting was performed to assess the interaction between ROBO1 and LRP6.

### Statistics.

All experiments were performed at least 3 times independently, with representative results shown. All data were analyzed using GraphPad Prism 10.0 software and are expressed as mean ± standard deviation (SD). Comparisons between 2 groups were conducted using a 2-tailed Student’s *t* test. Correlations between SLIT2 concentration and TMJOA grading were assessed using Spearman’s rank correlation. For comparisons involving more than 2 groups, 1-way ANOVA followed by Tukey’s or Dunnett’s multiple-comparison test, or 2-way ANOVA with Šidák’s post hoc test, was applied as appropriate. If data did not meet the assumptions of normality, nonparametric statistical tests were employed. During the analysis, group allocations were blinded to ensure unbiased assessment. Statistical significance was determined at a threshold of *P* less than 0.05.

### Study approval.

The human TMJOA experiments were approved by the Ethics Committee of the Affiliated Stomatological Hospital of Guangzhou Medical University (JCYJ2024001). The human knee OA study was approved by the Ethics Committee of the Guangdong Provincial People’s Hospital (XJS2023-021-01). The animal experimental procedures were approved by the Ethics Committee of the China Microbiology Testing Center (202211002).

### Data availability.

All data are included in the Supporting Data Values file. The data presented in this study are available upon request. To facilitate access, please include a brief statement outlining the intended use of the data. The RNA-seq raw data were deposited to the National Genomics Data Center (NGDC) (HRA010725).

## Author contributions

CL and WJZ designed and conducted the experiments. GL, BC, and WC performed most of the in vivo and in vitro experiments and wrote the manuscript. HL and WG assisted with some in vivo experiments and data analysis. QZ, JL, LW, and JLP contributed reagents or materials. YY, WZ, XZ, and BZ performed the bioinformatics analyses. ZW, SW, and JH reviewed the manuscript. The manuscript was approved after all authors reviewed and confirmed the data. The order of co–first authorship was determined based on overall contribution and was agreed upon by all authors.

## Funding support

- National Natural Science Foundation of China grant 82370985 (to QZ).
- National Key Research and Development Program of China grant 2021YFE0108000 (to JL).
- Natural Science Foundation of Guangdong Province grant 2026A1515012594 (to CL).
- Guangzhou Science and Technology Plan Project 2023A03J0327 (to CL).

## Supplementary Material

Supplemental data

Unedited blot and gel images

undefined

Supporting data values

## Version 1 04/08/2026

Electronic publication
