# Supplementary material for: Activation of SLIT2/ROBO1/LRP6 axis aggravates cartilage degradation via β-catenin signaling in TMJOA
Source: JCI Insight. 2026 Apr 8;11(7):e193632. doi: 10.1172/jci.insight.193632 (PMC13134713; doi:10.1172/jci.insight.193632)
Supplement: Supplemental data [file jciinsight-11-193632-s242.pdf]

Supplemental PDF

Activation of SLIT2/ROBO1/LRP6 axis aggravates cartilage degradation via beta-catenin signaling in TMJOA.

Guan Luo[1, #], Baoyi Chen[1, #], Wenjun Chen[1, #], Huiyi Lin[1,2], Weiqi Guo[1,3], Qingbin Zhang[1], Jiang Li[1], Lijing Wang[4], Janak Lal Pathak[1], Yuhui Yang[5], Weijun Zhang[1], Xiaoyu Zhang[1], Beining Zheng[1], Ziyi Wang[1], Shiting Wei[1], Jiaxin He[1], Wei-Jie Zhou[6, *], Chang Liu[1, *]

[1]Department of Orthodontics, School and Hospital of Stomatology, Guangdong Engineering Research Center of Oral Restoration and Reconstruction, Guangzhou Key Laboratory of Basic and Applied Research of Oral Regenerative Medicine, Guangzhou Medical University, Guangzhou, Guangdong, China.

[2]Department of Orthodontics, Jiangmen Municipal Stomatology Hospital, Jiangmen, Guangdong, China.

[3]Department of Stomatology, The First Affiliated Hospital of Guangdong Pharmaceutical University, Guangzhou, Guangdong, China.

[4]Vascular Biology Research Institute, School of Life Sciences and Biopharmaceutics, Guangdong Pharmaceutical University, Guangzhou, Guangdong, China.

[5]Department of Orthopedics, Guangdong Provincial People's Hospital (Guangdong Academy of Medical Sciences), Southern Medical University, Guangzhou, Guangdong, China.

[6]Department of General Surgery, Guangdong Provincial Key Laboratory of Precision Medicine for Gastrointestinal Tumor, State Key Laboratory of Multi-organ Injury Prevention and Treatment, Cancer Research Institute, School of Basic Medical Sciences, Nanfang Hospital, Southern Medical University, Guangzhou, Guangdong, China.

[#]These authors contributed equally to this work and shared the first authorship

[*]Correspondence to: Chang Liu, Department of Orthodontics, School and Hospital of Stomatology, Guangdong Engineering Research Center of Oral Restoration and Reconstruction & Guangzhou Key Laboratory of Basic and Applied Research of Oral Regenerative Medicine, Guangzhou Medical University Guangzhou, Guangzhou, Guangdong, China.

[*]Correspondence to: Wei-Jie Zhou, Department of General Surgery, Guangdong Provincial Key Laboratory of Precision Medicine for Gastrointestinal Tumor, State Key Laboratory of Multi-organ Injury Prevention and Treatment，Cancer Research Institute, School of Basic Medical Sciences, Nanfang Hospital, Southern Medical University, Guangzhou, Guangdong, China

E-mail addresses and telephone: changliudentist@gzhmu.edu.cn, +8613710210667 (Chang Liu); weijiezhouum@163.com, +8613925005634 (Wei-Jie Zhou).

Conflict of interest: The authors have declared that no conflict of interest exists.

Supplemental Figure

A

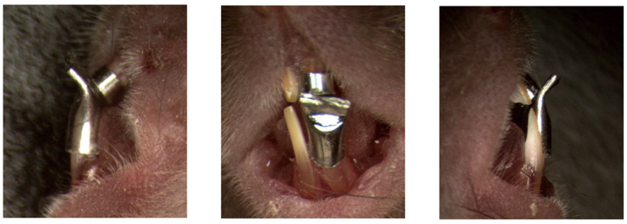

Supplemental Figure 1. (A) Frontal and lateral view images of a 6-week-old mouse following UAC induction.

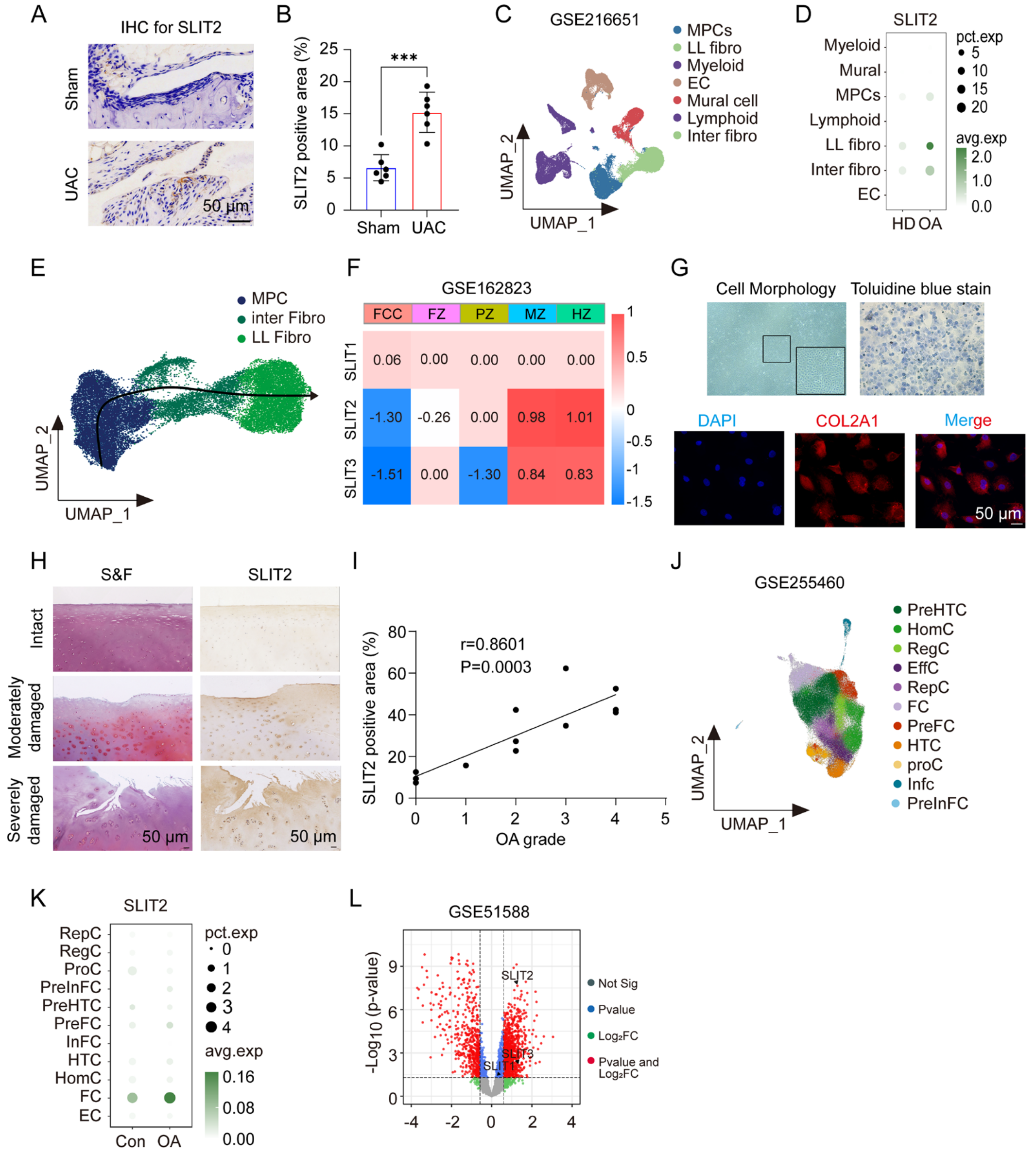

Supplemental Figure 2. (A-B) IHC for SLIT2 protein expression in TMJ synovium and quantitative image analysis (n = 6). (C) UMAP plot of cells from healthy human synovium and IPFPs based on scRNA-seq data from the GSE216651 dataset. (D) Distribution and expression of SLIT2 in different cell types. HD, Healthy donors; OA, osteoarthritis; MPCs, Mesenchymal progenitor cells; EC, Endothelial cells; Inter. fibro., Intermediate fibroblast; LL fibro., Synovial lining layer fibroblast. (E) Pseudotime trajectory mapping of MPCs was performed using the Slingshot algorithm. (F) Heatmap of SLIT family gene expression across different layers of condylar cartilage from the GSE162823 database. FCC, Femoral condylar cartilage; FZ, Fibrous zone; PZ, Proliferative zone; MZ, Mature chondrocytes zone; HZ, Hypertrophic chondrocytes zone. (G) Characterization of primary chondrocytes isolated from 3-week-old mice through cell morphology assessment, toluidine blue staining, and COL2A1 IF staining. (H) S&F and IHC show SLIT2 expression in the cartilage of knee OA patients at varying degrees of damage. (I) Correlation analysis between OA grade (OA grade assessment was based on the OARSI Osteoarthritis Cartilage Histopathology Assessment System) and the SLIT2 positive area. Data points

represent individual samples, and the line represents the best-fit linear regression. (J) UMAP plot of cells from three non-OA control donors and eight OA donors, based on scRNA-seq data from the GSE255460 dataset. RegC, Regulator chondrocytes (CHI3L1 and CHI3L2); ProC, Proliferation chondrocytes (C11orf96 , BMP2 and HMGA1); HomC, Homeostasis chondrocytes (HSPA1B , HSPA1A , HSPA6 , DDIT3 and JUN); preHTC, Prehypertrophic chondrocyte (PRG4 , ABI3BP and CRTAC1); EffC, Effector chondrocytes (CHRDL2, FRZB and CYTL1); FCs, Fibrocartilage chondrocytes (MMP2 , COL1A1 and COL1A2); HTC, Hypertrophic chondrocyte (SPP1, IBSP and COL10A1); RepC, Reparative chondrocytes (CILP2, CILP, and OGN); preFC, Prefibrocartilage chondrocyte (COL27A1, PLCG2 and WWP2); preInFC, Pre-inflammatory chondrocyte (IFI16 and IFI27); InfC, Inflammatory chondrocyte (CXCL8 , CD74 and GPR183). (K) Distribution and expression of SLIT2 in different chondrocyte subpopulations. Con, Control. (L) Volcano plot depicting the expression of SLIT2 genes in subchondral bone from OA and non-OA samples in the GSE51588 dataset. All data are shown as means ± SD. Statistical significance was assessed using two-tailed Student's t-test and Spearman's rank correlation. ***$P < 0.001$.

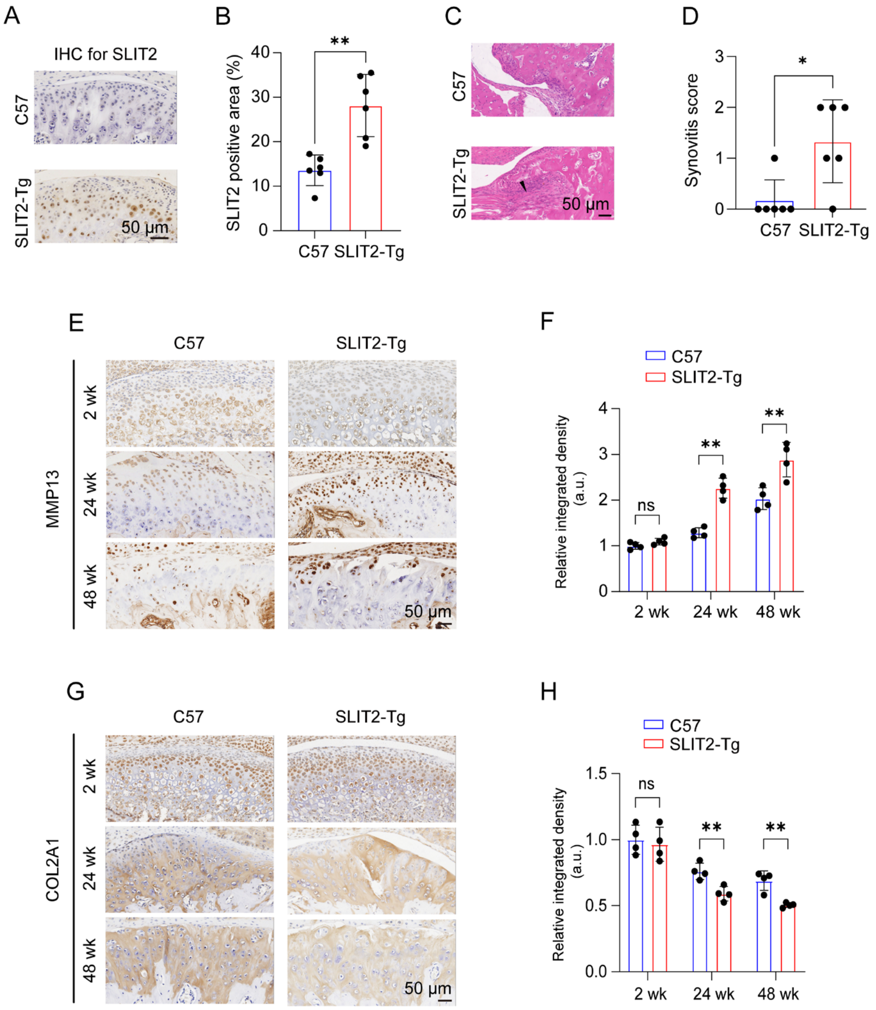

Supplemental Figure 3. (A-B) IHC analysis showing increased SLIT2 expression in the condylar cartilage of 10-week-old *SLIT2-Tg* mice, and quantitative analysis of the percentage of SLIT2 positive area (n = 6). (C-D) H&E showing that the synovium of *SLIT2-Tg* mice exhibited marked thickening and increased inflammatory cell infiltration compared with that of C57 mice, and quantitative analysis of synovitis score (n = 6). Synovitis severity was semi-quantitatively evaluated based on synovial lining thickening. Grade 0 represented normal morphology with 1–3 cell layers, grade 1 corresponded to 4–6 layers, and grade 2 indicated more than 7 layers (1). (E-F) IHC staining for MMP13 of 2-week, 24-week, and 48-week condylar cartilage, and quantitative analysis of integrated density (n = 4). (G-H) IHC staining for COL2A1 of 2-week, 24-week, and 48-week condylar cartilage, and quantitative analysis of integrated density (n = 4). All data are shown as means ± SD. Statistical significance was assessed using two-tailed Student's t-test or Mann–Whitney test. ns, not significant. **P* < 0.05, ***P* < 0.01, and ****P* < 0.001.

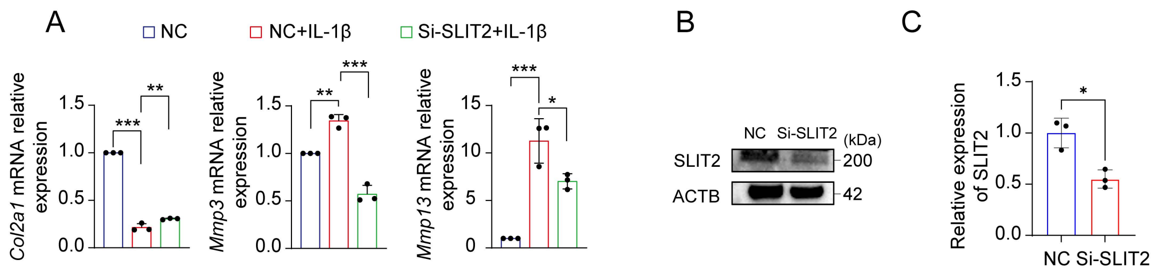

Supplemental Figure 4. (A) qRT-PCR analysis showing that SLIT2 knockdown reverses chondrocytes catabolism induced by 10 ng/mL IL-1B treatment in mouse primary chondrocytes for 24 h. (B-C) Western blot detection of SLIT2 knockdown in SW1353 chondrocytes, and relative quantification of SLIT2 proteins. All data are shown as means ± SD. Statistical significance was assessed by two-tailed Student's t-test and one-way ANOVA with Dunnett's multiple-comparison test. *$P < 0.05$, **$P < 0.01$, and ***$P < 0.001$.

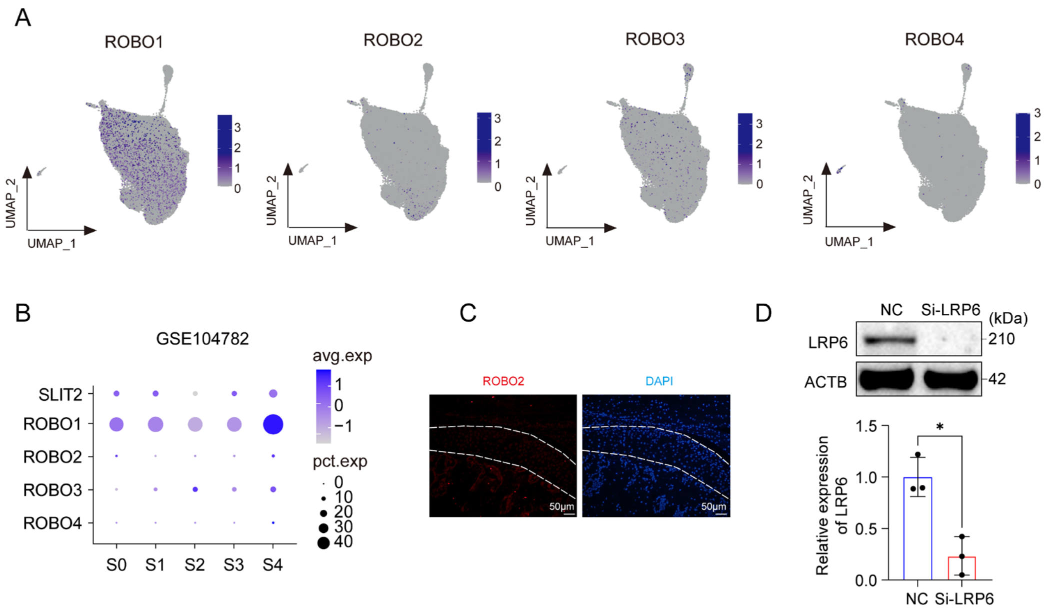

Supplemental Figure 5. (A) Feature plot visualization of expression and distribution patterns of ROBO1, ROBO2, ROBO3, and ROBO4 across various chondrocyte subpopulations from the GSE255460 dataset. (B) Expression of the SLIT2, ROBO1, ROBO2, ROBO3, and ROBO4 genes in human OA chondrocytes at different stages from single-cell dataset GSE104782. S0 and S1 (early-stage of OA; S3 and S4 (late-stage of OA). (C) IF showing ROBO2 localization in the condylar cartilage of 10-week-old mice. (D) Western blot detection of LRP6 knockdown in SW1353 chondrocytes, and relative quantification of LRP6 proteins. Statistical significance was assessed by two-tailed Student's t-test. *P<0.05.

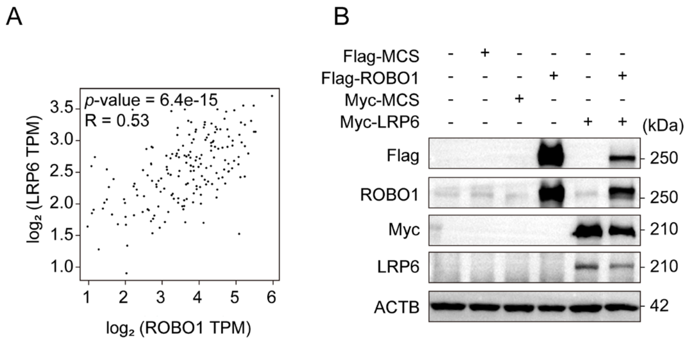

Supplemental Figure 6. (A) Correlation analysis between ROBO1 and LRP6 using the GEPIA (Gene Expression Profiling Interactive Analysis) platform reveals a potential relationship between the two genes. (B) Validation images of Flag-ROBO1 plasmid and Myc-LRP6 plasmid overexpression in SW1353 chondrocytes as indicated by Western blot.

Supplemental Table

	Sample	Age	Gender	Before treatment			After treatment	
				Imaging Scoring	Mouth opening (mm)	NRS score	Mouth opening (mm)	NRS score
1	Lavage fluid	31	female	4	25	2	49	0
2	Lavage fluid	29	male	4	42	2	42	0
3	Lavage fluid	20	male	4	33	3	33	0
4	Lavage fluid	16	female	4	31	3	40	1
5	Lavage fluid	41	female	4	44	1	45	0
6	Lavage fluid	40	female	4	45	2	45	0
7	Lavage fluid	33	female	4	31	1	34	1
8	Lavage fluid	23	male	4	45	0	45	0
9	Lavage fluid	59	female	4	30	4	32	2
10	Lavage fluid	15	female	4	35	1	45	0
11	Lavage fluid	48	female	4	45	1	45	0
12	Lavage fluid	25	female	3	30	3	40	0
13	Lavage fluid	52	female	3	24	2	30	0
14	Lavage fluid	15	female	3	42	2	44	0
15	Lavage fluid	51	male	3	45	1	45	0
16	Lavage fluid	20	female	3	33	1	39	0
17	Lavage fluid	21	female	2	43	3	48	1
18	Lavage fluid	28	female	2	26	3	40	0
19	Lavage fluid	31	female	2	46	1	49	0
20	Lavage fluid	20	female	2	50	3	50	0
21	Lavage fluid	44	male	2	40	2	45	0
22	Lavage fluid	27	female	2	27	2	35	0
23	Lavage fluid	28	female	2	25	3	25	2
24	Lavage fluid	15	female	2	38	3	52	0
25	Lavage fluid	27	female	2	44	2	44	0
26	Lavage fluid	25	female	2	45	2	45	0
27	Lavage fluid	49	female	2	45	1	50	0
28	Lavage fluid	19	female	1	40	4	40	2
29	Lavage fluid	24	male	1	37	3	48	0
30	Lavage fluid	16	female	1	45	3	54	0
31	Lavage fluid	39	female	1	30	1	32	0
32	Lavage fluid	29	female	1	32	4	37	1

33	Lavage fluid	24	female	1	47	2	47	0
34	Lavage fluid	27	female	1	26	3	31	1
35	Lavage fluid	27	female	1	50	0	50	0
36	Lavage fluid	16	female	1	28	0	42	0
37	Lavage fluid	15	female	1	50	4	50	2
38	Lavage fluid	26	male	1	45	1	55	0
39	Lavage fluid	26	female	1	45	2	48	0

Supplemental Table 1. **Volunteer information for TMJOA research.**

NRS score (2): Numerical rating scale: 0 painless; 1-3 Mild pain (pain does not affect sleep); 4-6 Moderate pain; 7-9 Severe pain (unable to fall asleep or awaken from pain during sleep); 10. Severe pain.

Radiographic Imaging Score for TMJOA (3)： Stage 1: The cortical bone of the condyle becomes indistinct, disappears, or presents with small concave defects. Stage 2: Extensive bone resorption and destruction occurred in the condyle. Stage 3: The areas of bone destruction in the condyle decreased, with evidence of repair. Stage 4: The condyle becomes shortened, with the anterior slope showing significant flattening and cystic changes. A new, intact cortical bone plate forms, often accompanied by flattening of the articular eminence and widening and shallowing of the glenoid fossa.

	Sample	Age	Gender	Side	Kellgren-Lawrence Grading
1	Knee joint	75	Female	Right	4
2	Knee joint	74	Female	Right	4
3	Knee joint	73	Female	Right	4
4	Knee joint	69	Female	Right	4
5	Knee joint	64	Female	Right	4

Supplemental Table 2. **Volunteer information for Knee OA research.**

Kellgren-Lawrence Grading for radiographic classification of knee osteoarthritis (4): Grade Description 0: Normal; 1: Questionable Doubtful narrowing of joint space and possible osteophytic lipping; 2: Mild Definite osteophytes and possible narrowing of joint space; 3: Moderate Moderate multiple osteophytes, definite narrowing of joint space, some sclerosis, and possible deformity of bone ends; 4: Severe Large osteophytes, marked narrowing of joint space, severe sclerosis, and definite deformity of bone ends.

	Sense	Anti-sense
Si-Slit2(Mus musculus)	5′- GCCCUAAAGUCAUCACCAATT -3′	5′- UUGGUGAUGACUUUAGGGCTT -3′
Si-SLIT2(Homo sapiens)	5′- GCAUCUGGUGUAAAUGAAATT-3′	5′- UUUCAUUUACACCAGAUGCTT -3′
Si-ROBO1(Homo sapiens)	5′- GCAACAUGGAGUGCUUACATT -3′	5′- UGUAAGCACUCCAUGUUGCTT -3′
Si-ROBO2(Homo sapiens)	5′- GCAGUCCACUGCAACUCUUTT -3′	5′- AAGAGUUGCAGUGGACUGCTT -3′
Si-ROBO3(Homo sapiens)	5′- GGCAGUCCUCCGUGAUGAUTT -3′	5′- AUCAUCACGGAGGACUGCCTT -3′
Si-ROBO4(Homo sapiens)	5′- CCAGGUCUGUACAGAUAUATT -3′	5′- UAUAUCUGUACAGACCUGGTT -3′
Si-LRP6(Homo sapiens)	5′- GGUGCUAACCGGAUAGUAUTT -3′	5′- AUACUAUCCGGUUAGCACCTT -3′

Supplemental Table 3. Target si-RNA sequences.

	Name	Forward	Reverse
**Human**	*COL2A1*	TGGACGCCATGAAGGTTTTCT	TGGGAGCCAGATTGTCATCTC
	MMP13	ACTGAGAGGCTCCGAGAAATG	GAACCCCGCATCTTGGCTT
	MMP3	AGTCTTCCAATCCTACTGTTGCT	TCCCCGTCACCTCCAATCC
	SOX9	AAGTCGGTGAAGAACGGG	TTTGGGGGTGGTGGGTGG
	ACAN	CCCCTGCTATTTCATCGACCC	GACACACGGCTCCACTTGAT
	SLIT2	AGCCGAGGTTCAAAAACGAGA	GGCAGTGCAAAACACTACAAGA
	GAPDH	TGACTTCAACAGCGACACCCA	CACCCTGTTGCTGTAGCCAAA
**Mouse**	*Slit1*	CACCATCGGACTCTTCACCC	CTGAACTCGCCCTTTTGCAC
	Slit2	AGCTTAGACGAATTGACCTGAGC	CCGAAGGCAGTTTATCTTGTTGG
	Slit3	GATCAACTGTCTGCGGGTGA	GCAGTCGCAAACAAATGGGT
	Robo1	CAACTCATCCAGGCGAACCT	AGACCCTCCTGTGTTCTGGT
	Robo2	GTGTCTGGGAAACGAAACGC	CTAGCTGCCACTTCTACCCG
	Robo3	GAATCGCCGAGAGGAACCAA	TTGCTGGGAGGAAAGTCTGC
	Robo4	CTTCCCCGCCAACAACCTAT	GTATCAGCGAGGAAGGAGCC
	Mmp13	CTTCTTCTTGTTGAGCTGGACTC	CTGTGGAGGTCACTGTAGACT
	Mmp3	ACATGGAGACTTTGTCCCTTTTG	TTGGCTGAGTGGTAGAGTCCC
	Col2a1	AAGGGACACCGAGGTTTCACTGG	GGGCCTGTTTCTCCTGAGCGT
	Gapdh	TGTGTCCGTCGTGGATCTGA	TTGCTGTTGAAGTCGCAGGAG

Supplemental Table 4. Primers for qRT-PCR.

Supplemental Table 5 (A standalone Excel file). The binding free energy between ROBO1 and LRP6 was predicted using the MM/GBSA method on the HawkDock platform (http://cadd.zju.edu.cn/hawkdock/). The total binding free energy was calculated to be -322.67 kcal/mol, indicating a strong interaction between the two proteins. Additionally, the per-residue energy contributions of the receptor are detailed in the accompanying table.

References for Supplemental Figure and Table.

1. Jariyasakulroj S, et al. Mapping cell diversity and dynamics in inflammatory temporomandibular joint osteoarthritis with pain at single-cell resolution. JCI Insight 2025;10(3).
2. Downie WW, et al. Studies with pain rating scales. Ann Rheum Dis 1978;37(4):378-381.
3. Xiaomin L, et al. [Comparison of three-dimensional position on cone beam computed tomography of temporomandibular joint osteoarthrosis]. Hua Xi Kou Qiang Yi Xue Za Zhi 2015;33(2):161-165.
4. Schiphof D, et al. Differences in descriptions of Kellgren and Lawrence grades of knee osteoarthritis. Ann Rheum Dis 2008;67(7):1034-1036.
