# Supplementary material for: Activation of SLIT2/ROBO1/LRP6 axis aggravates cartilage degradation via β-catenin signaling in TMJOA
Source: JCI Insight. 2026 Apr 8;11(7):e193632. doi: 10.1172/jci.insight.193632 (PMC13134713; doi:10.1172/jci.insight.193632)
Supplement: Unedited blot and gel images [file jciinsight-11-193632-s243.pdf]

Full unedited gel for Figure 1K

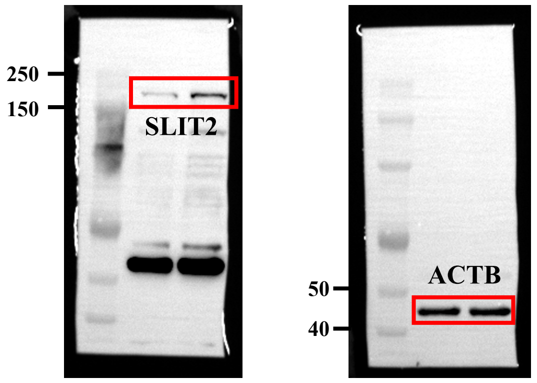

Full unedited gel for Figure 4A

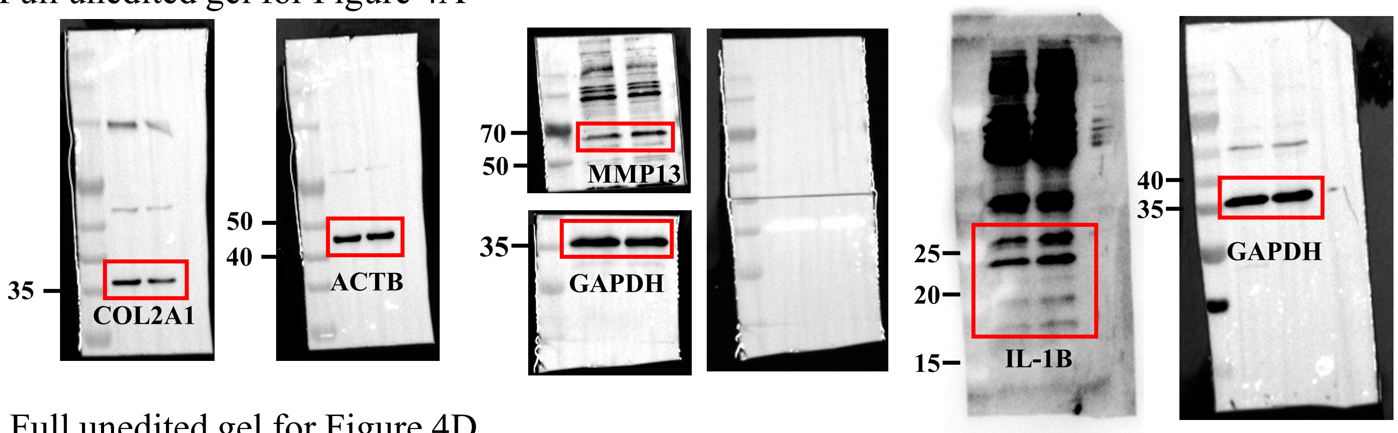

Full unedited gel for Figure 4D

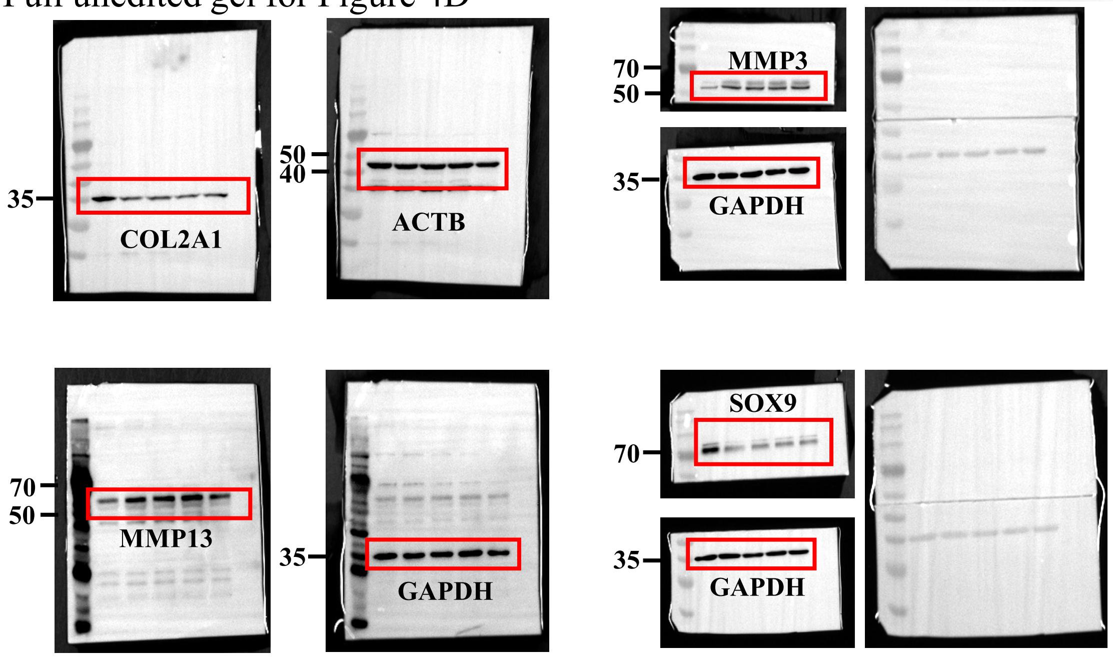

Full unedited gel for Figure 4F

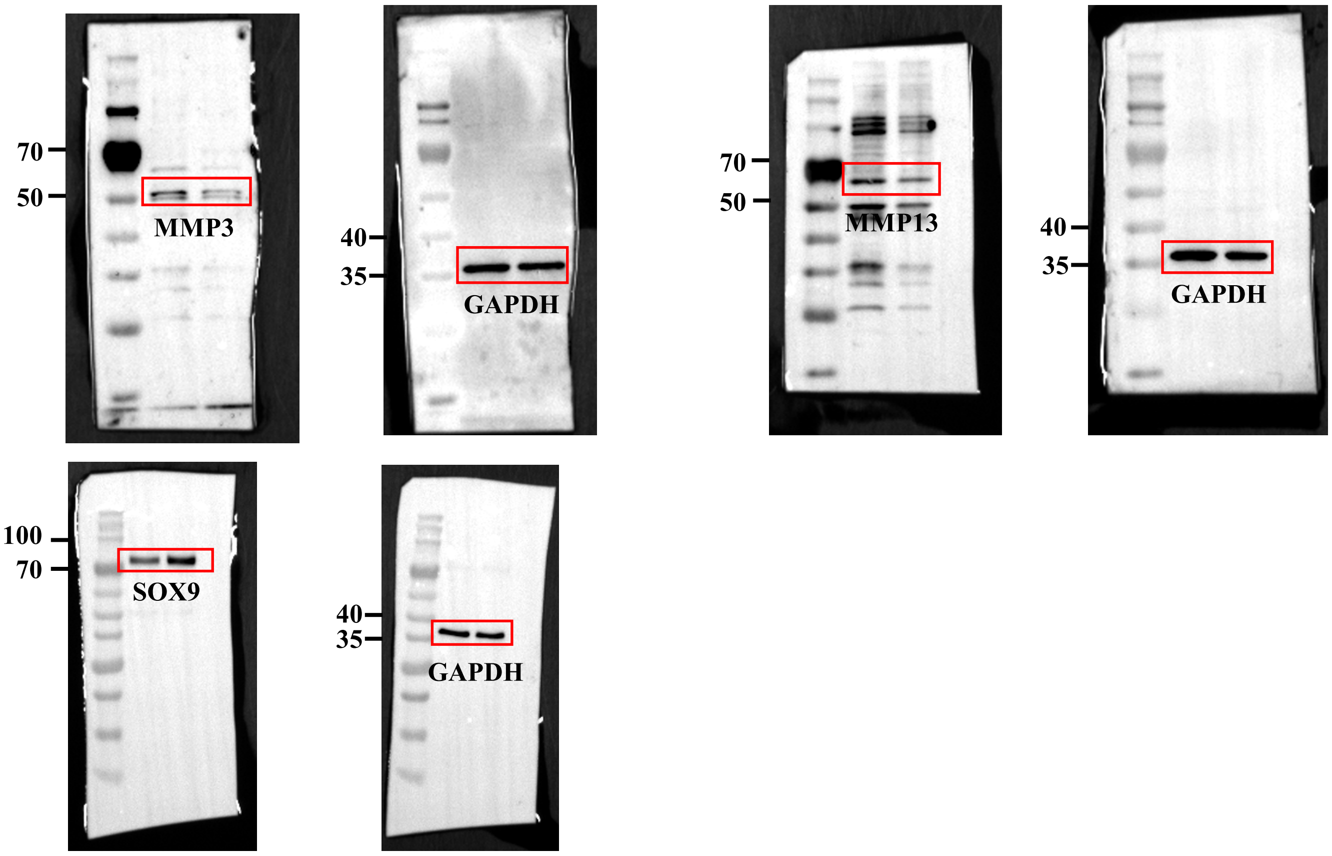

Full unedited gel for Figure 5B

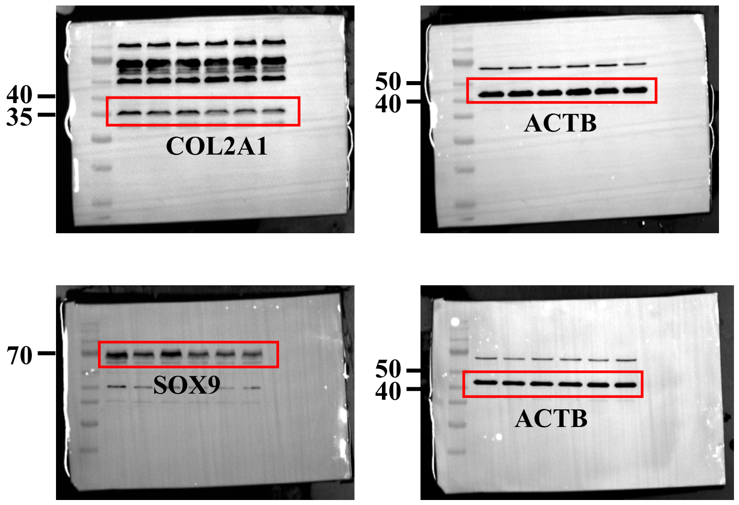

Full unedited gel for Figure 5N

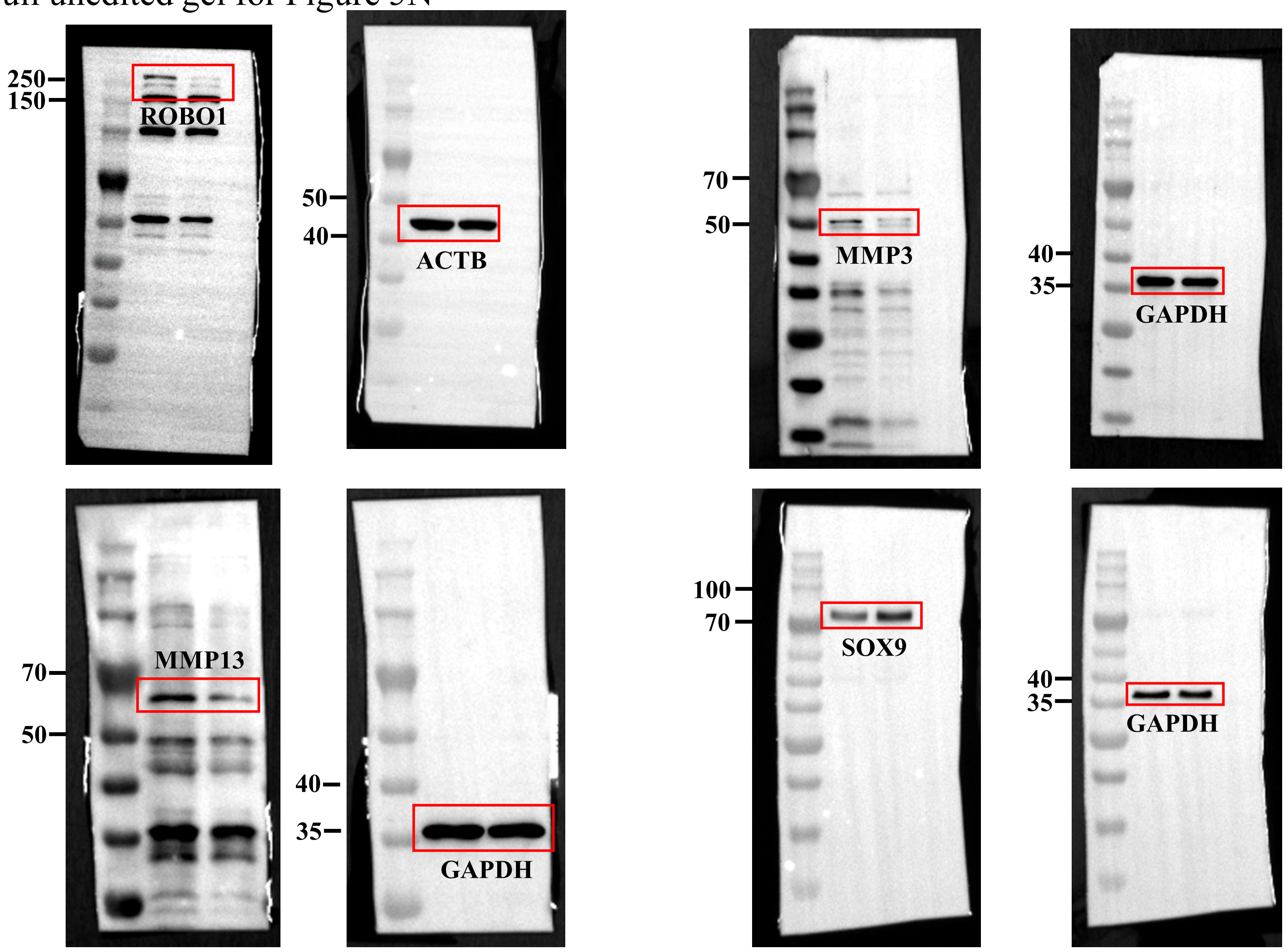

Full unedited gel for Figure 6F

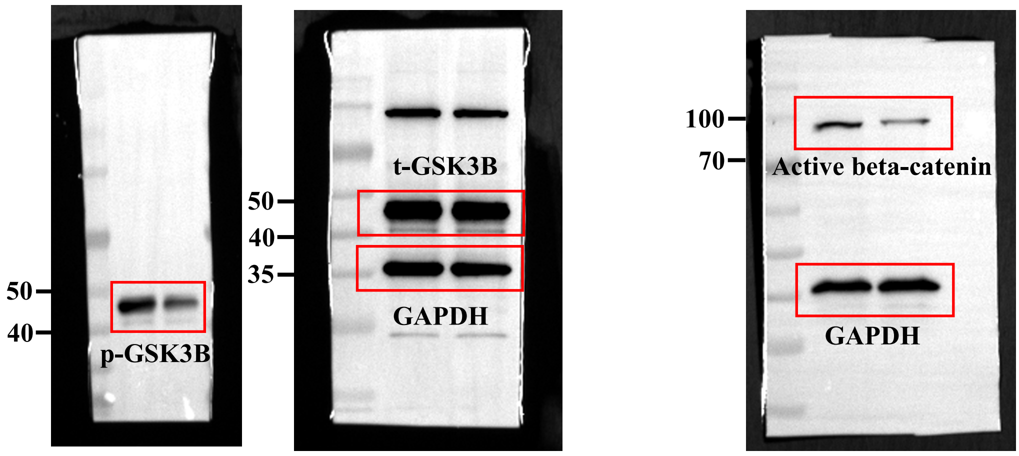

Full unedited gel for Figure 6J

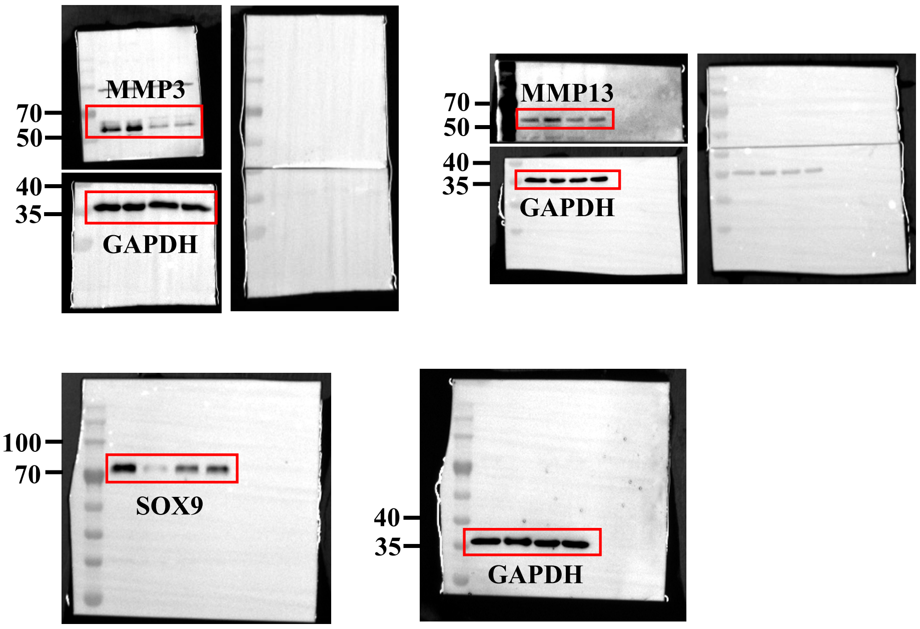

Full unedited gel for Figure 6L

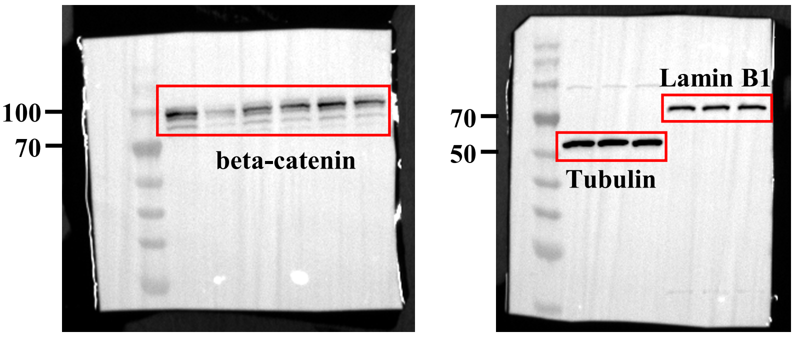

Full unedited gel for Figure 7A

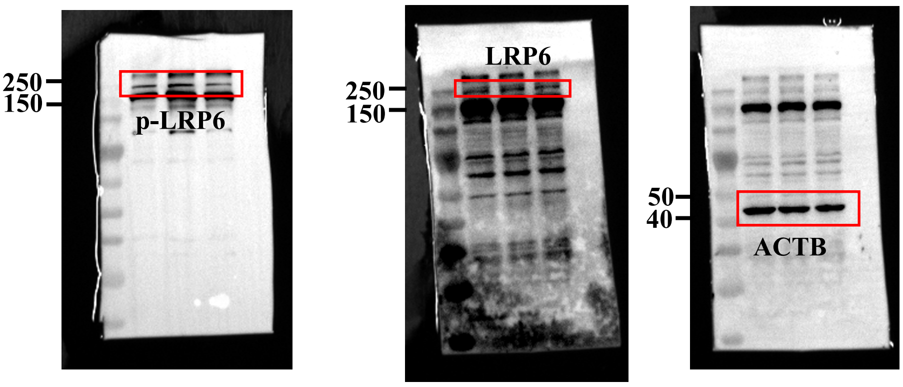

Full unedited gel for Figure 7E

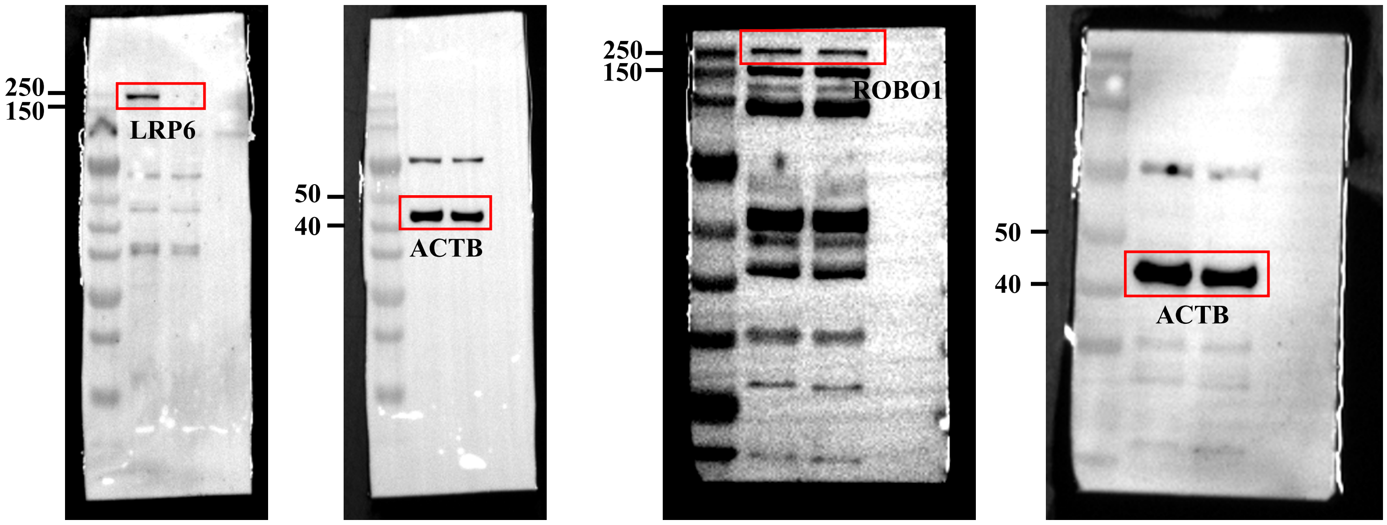

Full unedited gel for Figure 7H

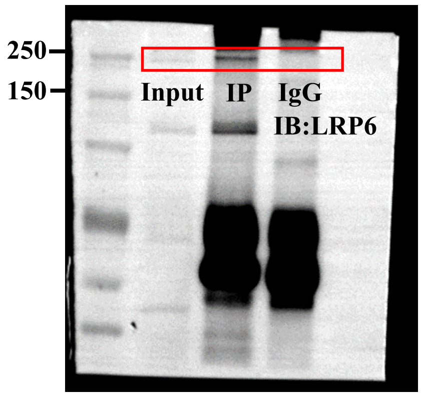

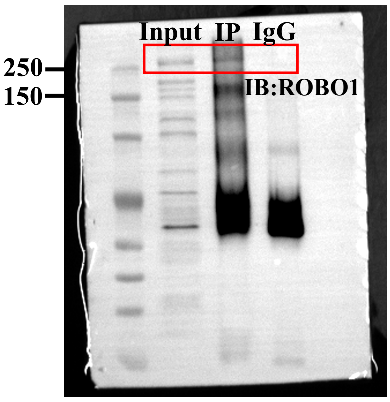

Full unedited gel for Figure 7I

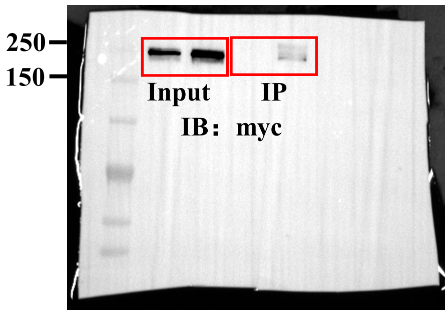

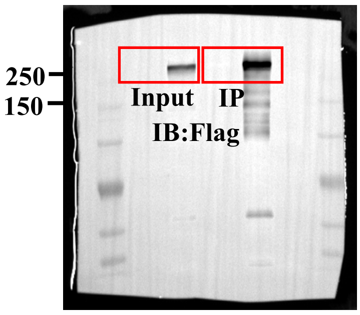

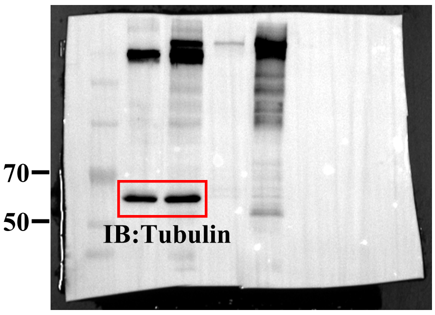

Full unedited gel for Figure 7J

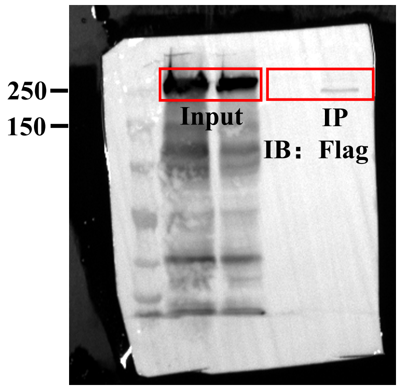

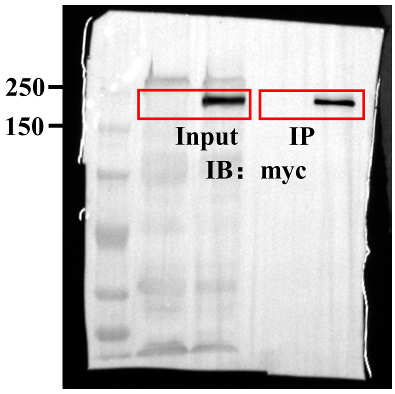

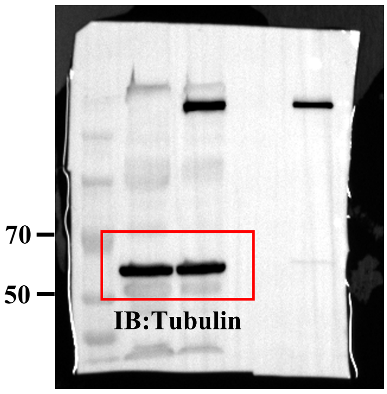

Full unedited gel for Figure 7K

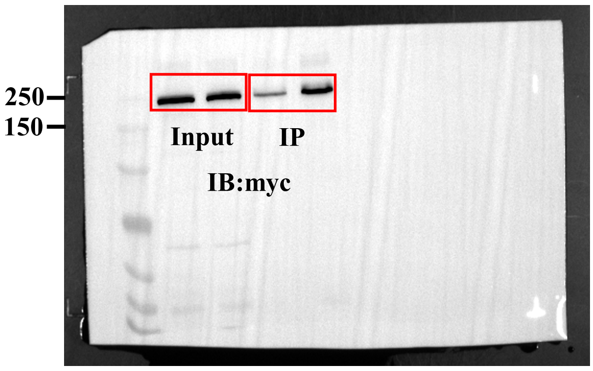

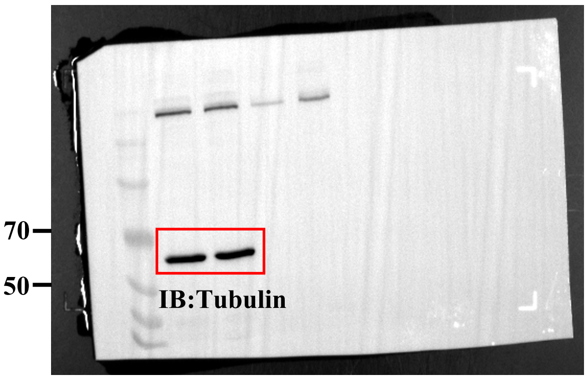

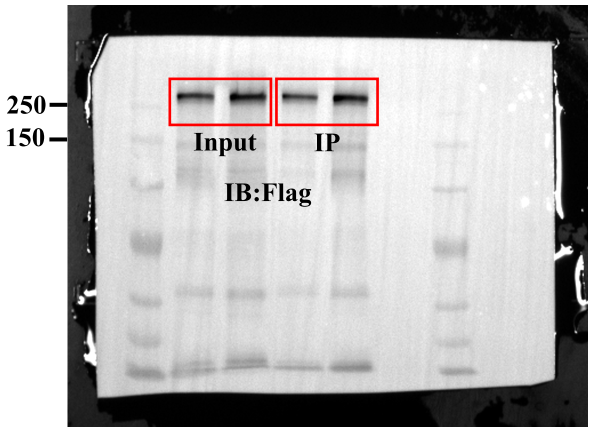

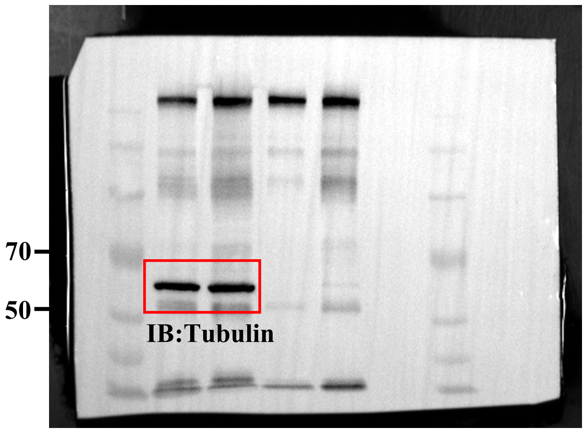

Full unedited gel for western blotting. Full unedited gel were detected by Tanon Imaging System (Beijing, China).
